# Predictors of 1-Year Mortality Among Patients With Heart Failure With Preserved Ejection Fraction

**DOI:** 10.1016/j.jacadv.2026.102847

**Published:** 2026-06-17

**Authors:** Fares Alahdab, Jack Lopuszynski, Mohammad Alkhateeb, Christopher Scott, Maliha Zahid

**Affiliations:** a Division of Cardiovascular Medicine, Department of Biomedical Informatics, Biostatistics, & Medical Epidemiology, and Department of Medicine, University of Missouri, Columbia, Missouri, USA; b Center for Women’s Health and Reproductive Medicine, Perelman School of Medicine, University of Pennsylvania, Philadelphia, Pennsylvania, USA; c Department of Medicine, University of Missouri, Columbia, Missouri, USA; d Department of Biostatistics, Mayo Clinic, Rochester, Minnesota, USA; e Department of Cardiovascular Medicine, Mayo Clinic, Rochester, Minnesota, USA

**Keywords:** albumin, HFpEF, mortality, nt-proBNP, prognosis, risk assessment

## Abstract

**Background:**

Accepted heart failure (HF) with preserved ejection fraction (EF) prognostic scores rely on limited variables and linear assumptions that are likely to miss complex risk patterns.

**Objectives:**

The objectives of the study were to develop, compare, and internally validate prediction models for 1-year all-cause mortality after first hospitalization for decompensated HF with preserved EF.

**Methods:**

We performed a retrospective cohort study using electronic medical records from a large academic health system, including adults with EF ≥50% admitted for a first-time HF exacerbation. Variables spanned demographics, comorbidities, laboratory tests, echocardiographic variables, medications, and outcomes. Data were split into training (80%) and test (20%) sets with stratification by outcome. Missing values were handled with multiple imputation by chained equations. Two tree-based classifiers (Extreme Gradient Boosting and Light Gradient Boosting) were tuned with cross-validation and evaluated by area under the receiver operating characteristic curve (AUROC) and calibration. Time-to-event models included Cox proportional hazards, random survival forest (RSF), and gradient boosting survival (GBS) with concordance index and calibration assessment. Global and local (patient-level) explainability was extracted from each model, with cross-model predictor ranking and comparison.

**Results:**

We analyzed 7,840 admissions; the mean age was 78 years with 55.6% women. One-year mortality was 31.5%. Test-set AUROC was 0.751 (95% CI: 0.727-0.775) for Extreme Gradient Boosting and 0.749 for (95% CI: 0.721-0.776) Light Gradient Boosting with acceptable calibration. GBS achieved the highest concordance index (0.718; 95% CI: 0.696-0.740), followed by RSF (0.711; 95% CI: 0.690-0.734) and Cox (0.704; 95% CI: 0.680-0.728). The 12-month time-dependent AUROCs for survival models were GBS 0.759 (95% CI: 0.716-0.799), RSF: 0.750 (95% CI 0.708-0.789), and Cox: 0.735 (95% CI 0.692-0.777). Lower albumin, older age, higher N-terminal pro-B-type natriuretic peptide, renal dysfunction, and lower hemoglobin were the most consistent risk signals.

**Conclusions:**

Our transparent risk tool using routinely available admission data appears feasible, allowing for patient-level, precision health risk assessment.

The prevalence of congestive heart failure (HF) continues to rise in the United States, with 6.7 million HF patients as of 2023, a number which is expected to rise to 8.5 million by 2030,^1^ with 1 in four Americans developing it in their lifetime. HF is categorized into HF with reduced ejection fraction (EF), HF with mildly reduced EF, and HF with preserved EF (HFpEF), which has steadily increased in prevalence due to risk factors like obesity, hypertension, and diabetes.^1^ HFpEF is characterized by metabolic dysfunction and inflammation, leading to fibrosis and an inability of the left ventricle to relax. Despite its prevalence, HFpEF has few therapies target its underlying pathophysiology,^2^ with treatment emphasizing symptom management; physicians use diuretic agents to treat congestion, treat comorbidities such as atrial fibrillation and coronary artery disease, and suggest lifestyle changes such as weight loss.^3^ Therefore, as treatments are developed for HFpEF, understanding the risk factors and prognosticators for this syndrome is of utmost importance both in designing future clinical trials and in the decision-making process during clinical treatment. Traditional risk models and clinical scores such as MAGGIC and the Seattle Heart Failure Model incorporate a limited set of clinical variables and rely on linear assumptions that may not accurately reflect HFpEF prognosis, as advances in biomarker research underscore the prognostic significance of broader-ranging molecules, including markers of myocardial fibrosis and renal dysfunction.^4^, ^5^, ^6^, ^7^ In parallel, capturing nonlinear relationships and handling multidimensional data is a frequent challenge with most risk assessment and prognostication models, particularly for a complex syndrome such as HFpEF.^4^^,^^8^ In this study, we use machine learning techniques for risk assessment of 1-year mortality in HFpEF patients, accounting for higher-dimensional data and nonlinear relationships in a large cohort of patients.

## Methods

This is a retrospective cohort study conducted and reported in accordance with the Strengthening the Reporting of Observational Studies in Epidemiology (STROBE) guideline.^9^ The machine learning models’ development and evaluation were conducted and reported in adherence to the Transparent Reporting of a multivariable prediction model for Individual Prognosis or Diagnosis (TRIPOD + AI) guideline.^10^

### Study design and cohort selection

We conducted a retrospective study to develop and validate a machine learning model for predicting 1-year all-cause mortality among patients with a first hospitalization for decompensated HFpEF. We queried Mayo Clinic electronic medical records from 3 sites (Rochester, Minnesota; Jacksonville, Florida; and Scottsdale, Arizona) for all adult patients (age ≥18 years) admitted between January 2010 and December 2020 with a primary diagnosis of HF exacerbation with left ventricular EF of >50% on echocardiography performed within 6 months before admission or up to 7 days postdischarge. The study was approved by the Mayo Clinic IRB, with a waiver of informed consent.

### Data collection and variables

Clinical data were extracted from electronic medical records. Variables included demographics at time of index hospitalization, comorbidities, laboratory values (including serum albumin measured on the day of admission or within 7 days, N-terminal pro-B-type natriuretic peptide (NT-proBNP), high-sensitivity cardiac troponin-T, renal function markers, and standard chemistry and hematology panels), echocardiographic parameters (including biatrial size, biventricular size and function, and estimated right ventricular [RV] systolic pressures), medications at discharge, and clinical outcomes including repeat hospitalization, time to repeat hospitalizations, and 1-year all-cause mortality. The primary outcome was all-cause mortality within 12 months of the index admission date. For time-to-event models, follow-up time was measured from the index admission date to death or censoring at 12 months (or last known contact). For binary classifiers, a fixed 12-month mortality indicator was used; patients alive or censored at 12 months were coded as 0, and patients who died within 12 months were coded as 1.

### Statistical analysis

Patient data were summarized using mean and SDs, or medians and IQR, or frequencies/proportions, as appropriate. All analyses were conducted using Python 3.10. Statistical and machine learning computations utilized: scikit-learn for preprocessing and evaluation metrics; Extreme Gradient Boosting (XGBoost) and Light Gradient Boosting Machine (LightGBM) for binary classification; scikit-survival for survival analysis including Cox proportional hazards, Random Survival Forest (RSF), and gradient boosting survival analysis (GBSA); SHapley Additive exPlanations (SHAP) for global explainability; Local Interpretable Model-agnostic Explanations (LIME) for local interpretability; and standard libraries including NumPy, pandas, matplotlib, and seaborn for data manipulation and visualization. Statistical significance was set at α = 0.05 for all hypothesis tests. All random seeds were fixed to ensure reproducibility.

### Data preparation and splitting

Data were randomly split into training (80%) and testing (20%) sets using stratified sampling based on the event indicator to maintain consistent event-to-censored ratios across both sets. This stratification allowed balanced representation of the binary outcome (1-year mortality) in both data sets, which in turn is critical for reliable model evaluation in imbalanced clinical datasets. Random seed was set to 42 for reproducibility across all analyses.

### Missing data imputation

Missingness was assessed (Supplemental Table 9) early and variables that were missing from 35% or more of the cohort were dropped. Missing data were handled using multiple imputation by chained equations, a robust iterative imputation approach with superior performance compared to simple imputation methods.^11^ Features were categorized as numerical (continuous or discrete with >10 unique values) or categorical (≤10 unique values). Numerical features were imputed using multiple imputation by chained equations with random forest regressors as the estimator, performing 10 iterations to achieve convergence. Random forests were chosen as the imputation estimator due to their ability to capture nonlinear relationships and interactions without requiring distributional assumptions. Categorical features were imputed using mode imputation. Critically, all imputation models were fit exclusively on training data and subsequently applied to the test set to prevent data leakage, to avoid overly optimistic performance estimates.^12^

### Machine learning model development

Two gradient boosting algorithms were developed to predict binary 1-year mortality events. XGBoost was implemented using the XGBoost library, employing a regularized gradient boosting framework. LightGBM) was implemented as an advanced alternative, utilizing leaf-wise tree growth for enhanced efficiency. Both models underwent hyperparameter optimization using RandomizedSearchCV with 10-fold stratified cross-validation on the training set. The search space included: number of estimators (100, 200, 300), maximum depth,^3^, ^4^, ^5^, ^6^ learning rate (0.01-0.1), subsample and feature sampling ratios (0.8-1.0), regularization parameters, and scale_pos_weight to address class imbalance. RandomizedSearchCV sampled 100 random combinations to balance computational efficiency with thorough exploration.^13^ Model performance was optimized using the area under the receiver operating characteristic curve (AUROC).

Time-to-event outcomes were analyzed using 3 complementary survival analysis methods appropriate for right-censored data. RSF extends random forests to survival outcomes by adapting tree-splitting criteria to maximize survival differences between nodes.^14^ GBSA combines gradient boosting with Cox's partial likelihood loss function.^15^ Cox proportional hazards regression was implemented as the benchmark clinical standard for time-to-event analysis. For the Cox model, features were standardized using StandardScaler, and L2 regularization (alpha = 0.1) was applied to reduce overfitting.^16^ RSF and GBSA models underwent hyperparameter optimization using RandomizedSearchCV with 5-fold stratified cross-validation and concordance index (C-index) as the optimization metric. The search space for these models included: number of estimators (100-400), maximum depth, minimum samples per split and leaf, feature sampling strategies, and regularization parameters. All models were implemented using the scikit-survival package.^17^

These 2 frameworks are complementary: binary classifiers provide fixed-horizon 12-month mortality probability estimates, whereas survival models provide time-varying risk estimates that appropriately account for variable follow-up and censoring.

For feature selection, we used all candidate predictors with model-specific regularization (eg, shrinkage/penalties; early stopping where applicable). As prespecified sensitivity analyses, we evaluated univariate prescreening (for classifiers: F-test or mutual information; for survival, univariate Cox) to select top-k features before model fitting. All screening was performed within the cross-validation loop (on training folds only) to avoid optimistic bias.^10^ For the Cox model, all 51 candidate predictors were prespecified before modeling based on clinical and biological relevance to HFpEF prognosis, and were not selected through data-driven screening. L2 regularization was applied to all candidate predictors simultaneously; this penalization approach reduces overfitting and attenuates multicollinearity among correlated clinical predictors.

### Model evaluation

Binary classification models were evaluated using AUROC, average precision, accuracy, balanced accuracy, F1-score, and confusion matrices on the held-out test set. Survival models were evaluated using the C-index, which measures the probability that the model correctly ranks predicted survival times for randomly selected patient pairs. C-index ranges from 0.5 (random) to 1.0 (perfect); values >0.7 are commonly cited as reflecting acceptable discrimination in prognostic models,^18^ although the optimal threshold is context and outcome dependent. Time-dependent area under the curve was computed at multiple time points to assess discrimination as a function of follow-up time.^19^ The 12-month time-dependent area under the curve is the primary discriminative estimate for survival models, consistent with the primary outcome. Model calibration was assessed graphically: reliability diagrams (mean predicted probability vs observed event rate across deciles) for binary classifiers and comparison of predicted survival probabilities against Kaplan-Meier estimates at 12 months for survival models. No formal calibration hypothesis test was used, consistent with current guidance for large samples. Intermodel agreement was assessed using Pearson correlation coefficients, and pairwise independent t-tests compared model performance. Bootstrap resampling with 1,000 iterations computed 95% CIs for all performance metrics using the percentile method.

To compare AUROCs between classifiers on the same test set, we used the paired DeLong test.^20^ For differences in C-index between survival models, we used a paired, stratified bootstrap (1,000 replicates) to obtain ΔC with percentile CIs. We did not use independent t-tests for correlated performance measures.

### Model explainability and interpretability

SHAP provided global model interpretability by computing Shapley values from cooperative game theory.^21^ TreeExplainer was used for tree-based models, offering exact Shapley value computation.^22^ SHAP analyses included summary plots displaying feature importance and directional effects, bar plots showing mean absolute SHAP values, dependence plots illustrating feature relationships and interactions, and waterfall plots for individual predictions. LIME provided complementary instance-level explanations by fitting local linear approximations around individual predictions.^23^ LIME explanations were generated for representative patients across the risk spectrum (high-risk, median-risk, low-risk) using 15 features and 5,000 perturbations per instance. For survival models lacking native feature importance metrics, permutation importance was computed by measuring the decrease in C-index after randomly shuffling each feature.^24^

## Results

### Cohort and outcome

From 2010 to 2020, we identified 7,840 unique first admissions for HF exacerbation among HFpEf patients. The mean age was 78 years (IQR: 68-86), 55.6% were women, and 94.5% self-identified as Caucasian. Hypertension was common (86.1%), followed by hyperlipidemia (71.8%) and diabetes (53.9%). Among those with available data, 76.3% had RV systolic pressure ≥35 mm Hg. The median admission laboratory values were: albumin 3.6 g/dL (IQR: 3.2-3.9), NT-proBNP 2,391 pg/mL (IQR: 964-5,169), and estimated glomerular filtration rate (eGFR) 55 mL/min/1.73 m^2^ (IQR: 37-74.5). Baseline characteristics, including demographics, comorbidities, and medications, are provided in Table 1, whereas echocardiographic and lab findings are shown in Supplemental Tables 1 and 2, respectively. During a 1-year follow-up, 2,466 deaths (31.5%) occurred, corresponding to an incidence rate of 3.8 deaths per 100 person-years; 95% CI: 3.6 to 3.9. Data were randomly split into training (n = 6,272; 80%) and test (n = 1,568; 20%) sets with similar mortality rates (31.4% vs 31.6%).

**Table 1:** Baseline Demographics, Comorbidities, and Medications at Discharge

Sex	
Female	4,362 (55.6%)
Male	3,478 (44.4%)
Race	
Asian	60 (0.8%)
Black	169 (2.2%)
Native American	24 (0.3%)
White	7,408 (94.5%)
Other	85 (1.1%)
Unknown	94 (1.2%)
Age (years)	
<50	312 (4.0%)
50-64	1,142 (14.6%)
65-79	2,834 (36.1%)
80-94	3,273 (41.7%)
>94	279 (3.6%)
Body mass index (kg/m^2^)	
<25	1,811 (23.1%)
25.0-29.9	2,023 (25.9%)
30.0-34.9	1,587 (20.3%)
35.0-39.9	1,041 (13.3%)
≥40	1,362 (17.4%)
Missing	16
Comorbidities	
CAD	1,635 (20.9%)
COPD	3,058 (39.0%)
DM	4,223 (53.9%)
HTN	6,754 (86.1%)
Hyperlipidemia	5,632 (71.8%)
TIA	2,112 (26.9%)
Medications at discharge	
Aldosterone antagonists	363 (4.9%)
Diuretic agents	4,008 (51.1%)
SGLT2 inhibitors	7 (0.1%)

Values are n (%).

CAD = coronary artery disease; COPD = chronic obstructive pulmonary disease; DM = diabetes mellitus; HTN = hypertension; PF = pulmonary fibrosis; SGLT2 = sodium-glucose co-transporter 2; TIA = transient ischemic attack.

### Predictor screening

Of 51 candidate predictors, 39 (76.5%) were associated with 1-year mortality by F-test. The strongest univariate associations with 1-year mortality by F-test and univariate Cox regression included albumin, age, NT-proBNP, renal failure, blood urea nitrogen (BUN), body mass index (BMI), hemoglobin (HGB)/hematocrit, and left ventricular end-diastolic dimension (LVEDD). Supplemental Table 3 shows the univariate Cox model output, each variable’s coefficients, and effect estimates, whereas Supplemental Table 4 shows the multivariate Cox model output.

### Binary event prediction (XGBoost and LightGBM)

**Figure 1:**
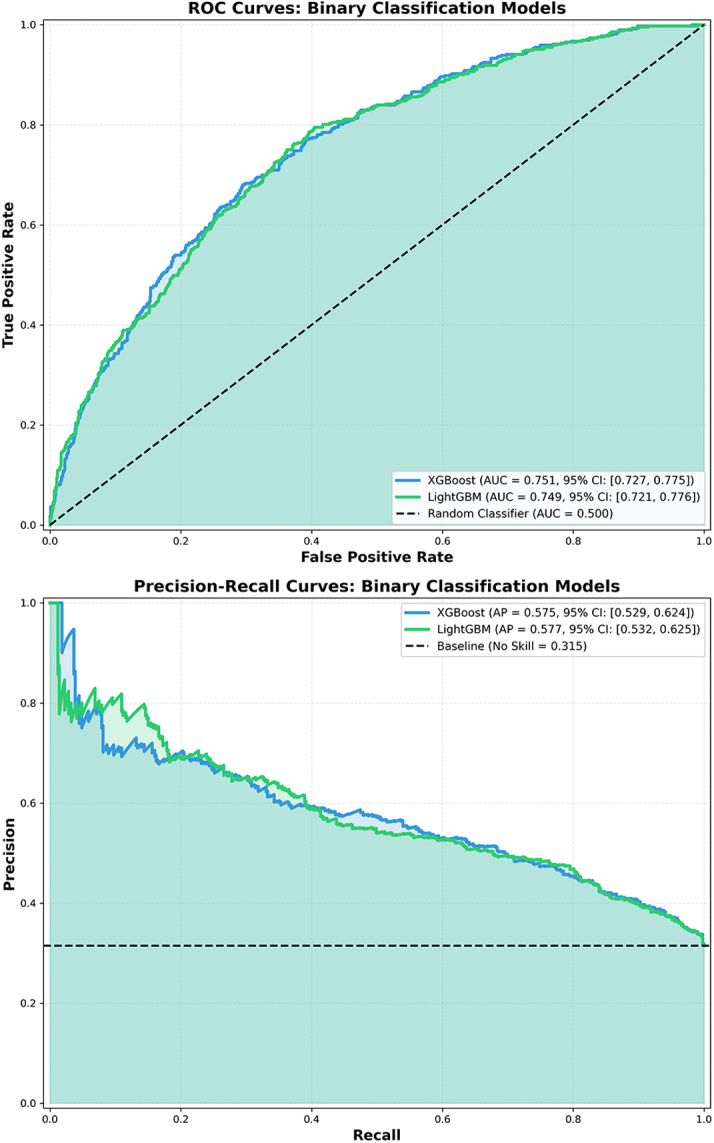
**Receiver Operating Characteristic and Precision-Recall Curves for the 2 Binary Classification Models (XGBoost and LightGBM)** Receiver operating characteristic (ROC) curves (Top) and precision-recall (PR) curves (Bottom) comparing XGBoost and LightGBM classifiers on the held-out test set (n = 1,568). Both models demonstrated moderate discrimination with overlapping performance: XGBoost achieved an area under the ROC curve (AUROC) of 0.751 (95% CI: 0.727-0.775) and LightGBM 0.749 (95% CI: 0.721-0.776). Average precision (AP) exceeded the no-skill baseline (0.315), confirming utility beyond class prevalence. The dashed diagonal line (Top) represents random classification (AUROC = 0.50); the horizontal dashed line (Bottom) represents the event-rate baseline. Shaded regions indicate 95% CIs. AUC = area under the curve; LightGBM = light gradient boosting machine; ROC = receiver operating characteristic curve; XGBoost = extreme gradient boosting.

Both boosted classifiers showed moderate discrimination on the held-out test set with overlapping CIs: XGBoost AUROC 0.751 (95% CI: 0.727-0.775); average precision 0.575 (95% CI: 0.529-0.624) and LightGBM AUROC 0.749 (95% CI: 0.721-0.776); AP 0.577 (95% CI 0.532-0.625). The AP values were well above the event-rate baseline (0.315). Discrimination and calibration are shown in Figure 1 (ROC and precision-recall curves) and Supplemental Figure 1 (calibration); calibration was close to the 45° line with mild underprediction at very low risk. Predicted probabilities from the 2 models were highly concordant (r = 0.962) (Supplemental Figure 1).

Regularized all-feature models outperformed univariate prescreened variants (eg, XGBoost-MI-25; LightGBM-F-test-25), so the regularized models are primary. A paired bootstrap comparison of AUROCs yielded a very small mean difference (∼0.002) with a 95% CI spanning zero; we therefore interpret no clear performance difference (Supplemental Table 5).

### Time-to-event prediction (Cox, RSF, and GBS)

**Figure 2:**
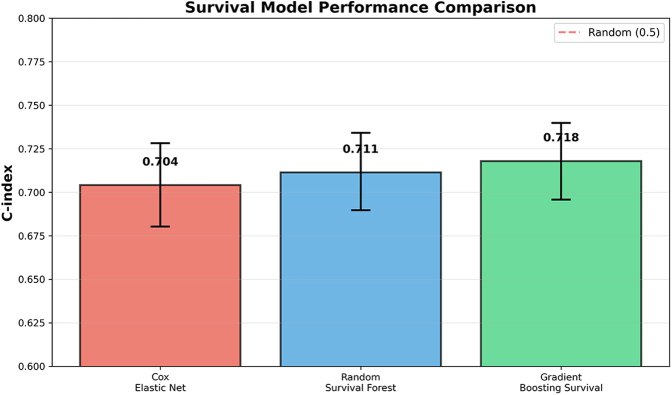
**C-Index Comparison (Time-to-Event Prediction) for the Cox Elastic Net, Random Survival Forest, and Gradient Boosting Survival Models** Bar plot comparing concordance index (C-index) across 3 time-to-event models evaluated on the test set. Gradient boosting survival (GBS) achieved the highest discrimination (C-index = 0.718), followed by random survival forest (RSF) (0.711) and Cox Elastic Net (0.704). Error bars represent bootstrap 95% CIs (1,000 iterations). Pairwise comparisons confirmed statistically significant differences (GBS vs Cox: ΔC = 0.014). All models exceeded random prediction (C-index = 0.50, indicated by red dashed line), demonstrating clinically useful prognostic accuracy for 1-year mortality prediction in heart failure with preserved ejection fraction.

Among survival models, GBS had the highest discrimination, followed by RSF and Cox Elastic Net (Figure 2): GBS C-index 0.718 (95% CI: 0.696-0.740); RSF 0.711 (95% CI: 0.690-0.734); and Cox 0.704 (95% CI: 0.680-0.728). The 12-month time-dependent AUROCs for survival models were: GBS 0.759 (95% CI: 0.716-0.799); RSF 0.750 (95% CI: 0.708-0.789); and Cox 0.735 (95% CI: 0.692-0.777). Pairwise bootstrap comparisons favored GBS > RSF (ΔC 0.0062; 95% CI: 0.0052-0.0071) and RSF > Cox (ΔC 0.0076; 95% CI: 0.0065-0.0085); GBS > Cox (ΔC 0.0137; 95% CI: 0.0127-0.0147) (Supplemental Table 6). Calibration at 12 months demonstrated good agreement between predicted and Kaplan-Meier observed event probabilities across deciles for all 3 survival models, with predictions confined to the 0% to 35% range consistent with the overall event rate (Supplemental Figure 2). Cox Elastic Net showed the closest adherence to the diagonal (mean calibration error [MCE] = 0.014), followed by GBS (MCE = 0.020) and RSF (MCE = 0.024) Preselection to a reduced feature set lowered performance for RSF/GBS, so all-feature fits are primary.

### Independent risk and protective factors (multivariable Cox)

**Figure 3:**
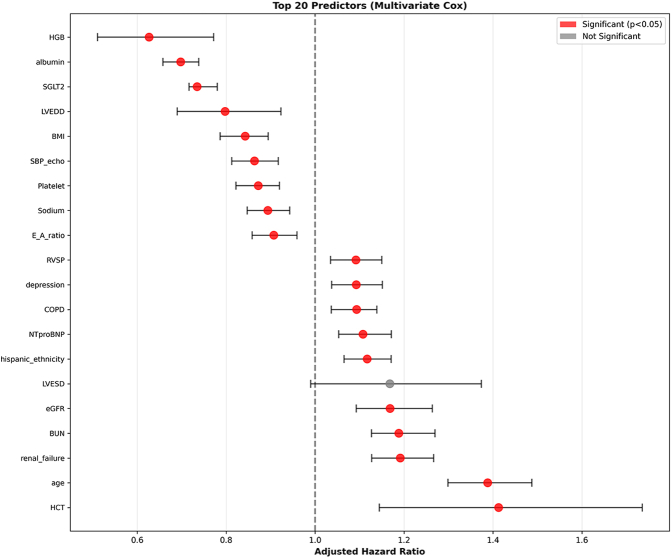
**Top 20 Multivariate Predictors and the HRs (95% CIs) for Each Predictor** Forest plot displaying adjusted HRs with 95% CIs for the top 20 independent predictors of 1-year mortality. Red circles indicate statistically significant associations (*P* < 0.05); asterisks denote significance. The vertical dashed line represents HR = 1.0 (no effect). Higher hematocrit (HCT; HR: 1.41), age (HR: 1.39), and renal failure (HR: 1.19) were associated with increased mortality, while higher hemoglobin (HGB; HR: 0.63), albumin (HR: 0.70), and SGLT2 inhibitor use (HR: 0.73) were protective. Horizontal lines represent 95% CIs. E_A_ratio = mitral E/A ratio; LVESD = left ventricular end-systolic dimension; SGLT2 = sodium-glucose co-transporter 2.

In the fully adjusted model, several variables remained independently associated with higher 1-year mortality (Figure 3, Supplemental Table 3). The strongest associations were observed for hematocrit (HR: 1.41; 95% CI: 1.16-1.77), age (HR: 1.39; 95% CI: 1.30-1.49), renal failure (HR: 1.19; 95% CI: 1.13-1.26), BUN (HR: 1.19; 95% CI: 1.12-1.27), and NT-proBNP (HR: 1.11; 95% CI: 1.05-1.17).

Conversely, several variables were independently protective after adjustment (Figure 3, Supplemental Table 3). Higher HGB (HR: 0.63; 95% CI: 0.51-0.76) and higher albumin (HR: 0.70; 95% CI: 0.66-0.74) were associated with lower risk, consistent with the explainability analyses. Sodium-glucose co-transporter 2 (SGLT2) inhibitor use was also associated with lower 1-year mortality (HR: 0.73; 95% CI: 0.72-0.78). Additional independent protective associations included LVEDD (HR: 0.80; 95% CI: 0.67-0.91), higher BMI (HR: 0.84; 95% CI: 0.78-0.90), higher systolic blood pressures at time of echo (SBP_echo; HR: 0.86; 95% CI: 0.82-0.91), and platelet count (HR: 0.87; 95% CI: 0.82-0.92).

### Signal coherence across approaches

**Figure 4:**
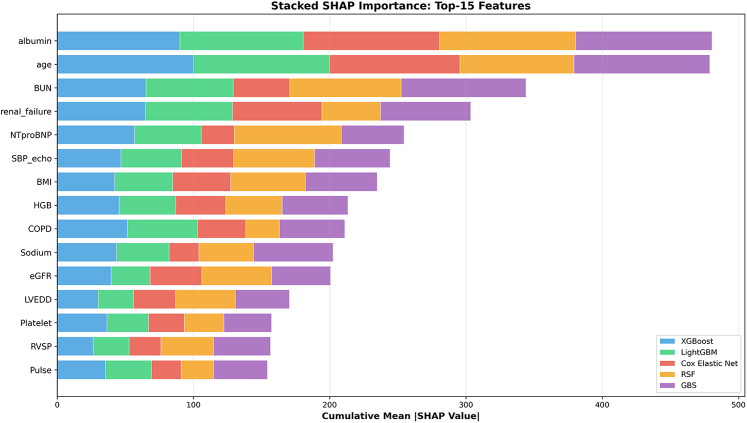
**Stacked SHapley Additive exPlanations Importance (Top-15)** Stacked horizontal bar plot displaying cumulative mean absolute SHapley Additive exPlanations (SHAP) values for the top-15 predictors across all 5 models. Albumin and age contributed the largest share of predictive importance, followed by blood urea nitrogen (BUN), renal failure, and N-terminal pro-B-type natriuretic peptide (NTproBNP). Color segments represent individual model contributions: XGBoost (teal), LightGBM (green), Cox Elastic Net (coral), random survival forest (RSF; purple), and gradient boosting survival (GBS; gold). The consistency of rankings across algorithm families demonstrates signal coherence and supports clinical interpretability. BMI = body mass index; COPD = chronic obstructive pulmonary disease; eGFR = estimated glomerular filtration rate; HGB = hemoglobin; LVEDD = left ventricular end-diastolic dimension; RVSP = right ventricular systolic pressure; SBP_echo = systolic blood pressure at echocardiography.

Across classification and survival frameworks, the leading predictors were consistent: lower albumin, older age, higher NT-proBNP, renal dysfunction markers (renal failure/BUN), lower HGB/hematocrit, and larger LVEDD were associated with higher risk; SGLT2-inhibitor use was associated with lower risk in univariate survival analysis (Figure 4, Supplemental Figures 3 to 6).

### Model explainability

#### Global patterns and cross-model stability

Per-model SHAP summary plots identified a stable set of high-impact predictors with consistent directionality (Figure 4, Supplemental Figures 4 to 12): albumin and age were top-ranked across all models, followed by BUN, renal failure, NT-proBNP, SBP_echo, BMI, HGB, eGFR, sodium levels, and LVEDD. Color gradients showed expected directions: lower albumin, older age, lower eGFR and higher NT-proBNP pushed predictions upward; higher SBP_echo and higher HGB tended to be protective.

Two cross-model visual summaries emphasize stability. The radar plot of the top-8 features (normalized 0-100) shows albumin levels and age near 100 for every model; survival tree/boosting models place relatively higher weight on BUN (highest in GBS) and NT-proBNP (highest in RSF), whereas penalized Cox down-weights these relative to trees (Supplemental Figure 5). The stacked top-15 plot shows the cumulative contribution across models; albumin + age account for the largest share, followed by BUN, renal failure, and NT-proBNP; the remainders form a longer tail (Figure 4).

The SHAP comprehensive statistics (Supplemental Table 7) confirm the average ranking for each variable: albumin, age, BUN, NT-proBNP, SBP_echo, BMI, and HGB. This feature consistency shows that each of these appeared in the top-20 of all 5 models (Supplemental Figures 7 to 12) with favorable average ranks. Intermodel agreement was high in the model-agreement heatmap (Supplemental Figure 6): SHAP-importance correlations were 0.994 (XGBoost-LightGBM) and 0.913 (RSF-GBS); the lowest pair, Cox-RSF, remained substantial (0.775).

#### Local patient-level explanations

LIME panels illustrate how predictors combine within individuals and mirror the global patterns (Supplemental Figures 13 to 17). In high-risk profiles, the largest positive weights concentrated on very old age (>86 yrs), low albumin, high BUN, renal failure, and very high NT-proBNP; median-risk cases showed mixed contributions (eg, low-normal albumin and renal impairment partly offset by other features); low-risk cases were characterized by preserved renal function (eGFR ≳74 mL/min/1.73 m^2^), higher albumin (>3.2 gm/dL), and SGLT2 therapy with low BUN and absence of RV abnormalities. The LIME summary table (Supplemental Table 8) aggregates these patterns across the binary models and patient risk strata.

## Discussion

In a contemporary HFpEF cohort, gradient-boosted classifiers achieved moderate discrimination for 1-year mortality (AUROC: ≈0.75) with acceptable calibration, whereas among survival models, gradient boosting survival performed best (C-index 0.718), followed by RSF and penalized Cox. Across algorithm families, the same clinical signals dominated risk: lower albumin (<3.2 g/dL), older age, higher NT-proBNP, and renal dysfunction (BUN/renal failure) consistently pointed to higher risk; SGLT2 inhibitor use was associated with lower risk in univariate survival analysis. These findings align with established epidemiology showing HFpEF in older, frequently female patients, with rising prevalence tied to population aging and cardiometabolic multimorbidity.^25^, ^26^, ^27^

Given the public health significance of HFpEF and its increasing prevalence, establishing prognostic models in a real-life patient population has direct implications for clinical care. These current models validate our previous work^28^ in a significantly larger, more diverse patient population. Our results extend prior HFpEF risk work by showing that modern tree/boosting methods and Cox agree on what matters, rather than uncovering new, model-specific signals. The repeated prominence of albumin and age across all models reinforces the prognostic value of nutritional/inflammatory status and biologic aging/frailty in HFpEF outcomes.^25^^,^^26^^,^^29^^,^^30^ In this study, an albumin level of <3.2 g/dL remained the single highest risk factor as both an independent predictor of prognosis with a HR of 2.006 (95% CI: 1.818-2.200) and as a multivariate predictor in both models including and excluding NT-proBNP (HR: 1.713; 95% CI: 1.535-1.920 and HR: 1.733; 95% CI: 1.551-1.915, respectively). Low albumin levels are being increasingly recognized as an adverse prognosticator in the setting of HFpEF by multiple investigators,^31^^,^^32^ correlating with longer hospital stays in males hospitalized for HF exacerbation,^33^ and associated with the development of HF in adults with baseline low albumin during 10 years of follow-up.^34^ Furthermore, low albumin remained a strong predictor of the primary endpoint in the TOPCAT trial even after adjustment for the MAGGIC risk score (HR: 0.72; 95% CI: 0.67-0.78; *P* < 0.0001).^30^ Similarly, in another cohort, albumin enhanced risk stratification beyond NT-proBNP and the MAGGIC score (adjusted standardized HR: 0.56; 95% CI: 0.37-0.83).^32^ The role of chronic systemic inflammation in the pathophysiology of HFpEF is being increasingly realized.^35^ Sustained elevation in tumor necrosis factor α and interleukin 6 levels suppress albumin production and increase capillary permeability, promoting albumin leakage into the interstitial space and urine,^36^^,^^37^ hence linking inflammation directly with low albumin in HFpEF and consequently adverse prognosis.

NT-proBNP has prognostic value despite known variability and multisystem influences,^5^^,^^38^^,^^39^ supporting its use as part of a cluster of markers rather than a stand-alone gatekeeper. Increased NT-proBNP levels are often associated with both the incidence and poor prognosis of HFpEF, a finding also supported by our data. After multivariable adjustment, NT-proBNP remained independently associated with higher 1-year mortality (adjusted HR: 1.11; 95% CI: 1.05-1.17). NT-proBNP’s value as a prognostic marker, however, is tempered by its susceptibility to elevation in multiple conditions, including peripheral artery disease, acute ischemic stroke,^40^ hypertension,^41^ COPD,^42^ atrial fibrillation,^43^ and more,^44^ as well as extremely large variations between patients.^5^ Indeed, in our study, NT-proBNP ranged from 5 to 65,900 pg/mL (mean 4,476.8 pg/mL; SD 6,240.2). An NT-proBNP ≥5,000 pg/mL corresponded to a materially higher unadjusted risk (≈22% relative increase). As expected, SGLT2 inhibitor use lowering mortality risk is consistent with randomized evidence in HFpEF/mid-range EF populations;^45^^,^^46^ we do not infer causality from our observational signal but note concordance with well-established trial data.

Global SHAP analyses showed high cross-model agreement (e.g., XGBoost-LightGBM r ≈ 0.99; RSF-GBS r ≈ 0.91), with albumin and age consistently top-ranked. Local (LIME) panels produced patient-level narratives that mirrored the global signals: very old age, low albumin, renal dysfunction, and markedly elevated NT-proBNP in high-risk profiles; preserved renal function, higher albumin, and SGLT2 therapy in low-risk profiles.^21^^,^^23^ These tools make the models auditable and clinically interpretable, whereas recognizing that associations within a fitted model do not represent causality.^47^^,^^48^

Two practical messages emerge. First, risk is driven by a recurrent cluster (albumin, age, renal indices, and NT-proBNP) of markers easily available on admission laboratory values. This supports routine risk stratification and targeted actions (nutrition support, renal-protective strategies, guideline-directed therapy, and SGLT2 eligibility checks) aligned with individual risk profiles.^25^^,^^45^^,^^46^ Second, because discrimination and calibration are somewhat similar across methods, health systems can adopt either a boosted classifier (for 1-year probability) or GBS/RSF (for time-to-event estimates) and focus operational effort on decision thresholds and net benefit. Consistent with reporting guidance, any deployment should include calibration monitoring and updating.^10^

### Study Limitations

Our study has several limitations. As this is an internally validated, single health system cohort study, the results should be regarded as exploratory and hypothesis-generating. External validation in demographically and geographically diverse populations is a prerequisite before any clinical implementation.^10^ The cohort was derived from 3 academic Mayo Clinic sites serving a predominantly Caucasian population (94.5%); incidence rates, comorbidity profiles, and clinical practices may vary substantially across community-based and safety-net healthcare settings in the United States, limiting generalizability. We relied on routinely collected data subject to coding and measurement error. We did not model cause-specific mortality or competing risks. Although explainability improved transparency, SHAP/LIME do not imply causality, and treatment decisions should continue to rely on trial-proven therapies.^45^^,^^46^^,^^48^ Finally, although all models demonstrated discrimination above C-index/AUROC >0.7, these values remain modest; individual-level predictions carry meaningful uncertainty and should be interpreted as decision-support outputs rather than deterministic classifiers. Future prospective evaluation with decision-curve analysis and prespecified decision-thresholds are needed to define clinical utility and implementation logistics.

## Conclusions

Across 5 modeling families, we observed convergent, clinically coherent predictors of 1-year mortality in HFpEF with consistent performance and acceptable calibration (Central Illustration). The dominance of albumin, age, renal indices, NT-proBNP, and the agreement of model-agnostic explanations, suggest that a transparent, implementable risk tool is feasible using data available at admission. External validation and prospective testing should precede routine use, but these results outline a clear foundation for risk-guided HFpEF care.

Perspectives **COMPETENCY IN SYSTEMS-BASED PRACTICE:**The comparable performance and calibration observed across boosted classifiers and survival-based models suggest that implementable risk prediction tools for HFpEF can be feasibly integrated into electronic health record systems. Such approaches could be utilized to support resource allocation, transitional care planning, and targeted population-health interventions in high-risk HFpEF populations.

**TRANSLATIONAL OUTLOOK:** Although the present models demonstrated moderate discrimination and consistent predictor stability across algorithmic approaches, external validation in geographically, racially, and socioeconomically diverse cohorts is required before clinical implementation. Prospective studies are needed to determine whether explainable machine learning models can improve clinician adoption, patient communication, and implementation of precision-guided therapeutic strategies in routine cardiovascular practice.

**Central Illustration:**
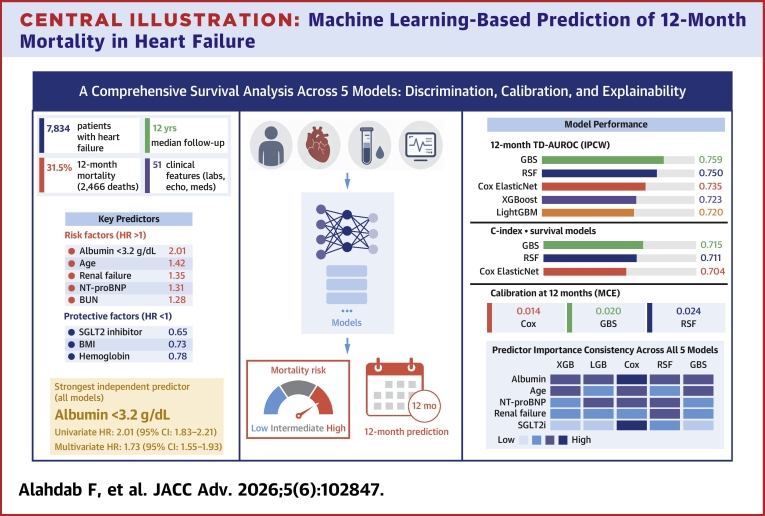
**Machine Learning-Based Prediction of 12-Month Mortality in Heart Failure** BUN = blood urea nitrogen; LightGBM = light gradient boosting machine; NT-proBNP: N-terminal pro-B-type natriuretic peptide; GBS = gradient boosting survival; MCE = mean calibration error; RSF = random survival forest; SGLT2i = sodium-glucose co-transporter 2 inhibitor; XGBoost = extreme gradient boosting.

## Funding support and author disclosures

Mr Lopuszynski and Dr Zahid are supported by 10.13039/100000002NIH grant R01HL153407 awarded to Dr Zahid. The authors have reported that they have no relationships relevant to the contents of this paper to disclose.
