# Supplementary material for: Predictors of 1-Year Mortality Among Patients With Heart Failure With Preserved Ejection Fraction
Source: JACC Adv. 2026 Jun 17;5(6):102847. doi: 10.1016/j.jacadv.2026.102847 (PMC13308253; doi:10.1016/j.jacadv.2026.102847)

| **Supplemental Table S1. Echocardiographic Findings at Index Hospitalization.** | |
| --- | --- |
| **Echocardiographic Parameter** | **N (%)** |
| Admission Rhythm (N=7311) |  |
| Atrial Fibrillation/Flutter | 2190 (30.0%) |
| Normal Sinus Rhyhtm | 5121 (70.0%) |
|  |  |
| Right Atrial Size (N=6998) |  |
| ≤Mild Enlargement | 3359 (48.0%) |
| Moderate Enlargement | 759 (10.8%) |
| Severe Enlargement | 1492 (21.3%) |
| Abnormal/Unknown Severity | 1388 (19.8%) |
|  |  |
| Left Atrial Size (N=7010) |  |
| ≤Mild Enlargement | 2804 (40.0%) |
| Moderate Enlargement | 925 (13.2%) |
| Severe Enlargement | 2075 (29.6%) |
| Abnormal/Unknown Severity | 1206 (17.2%) |
|  |  |
| Right Ventricular Size (N=7649) |  |
| ≤Mild Enlargement | 6053 (79.1%) |
| Moderate Enlargement | 941 (12.3%) |
| Severe Enlargement | 547 (7.1%) |
| Abnormal/Unknown Severity | 108 (1.4%) |
|  |  |
| Right Ventricular Pressure (N=6200) |  |
| <35 mmHg | 1467 (23.7%) |
| ≥35 mmHg | 4733 (76.3%) |
|  |  |
| Right Ventricular Function (N=7542) |  |
| ≤Mildly Reduced | 6408 (85.0%) |
| Moderately Reduced | 710 (9.4%) |
| Severely Reduced | 296 (3.9%) |
| Abnormal/Unknown Severity | 128 (1.7%) |
|  |  |
| Aortic Stenosis |  |
| ≤Mild | 7217 (92%) |
| Moderate | 339 (4.3%) |
| Severe | 284 (3.6%) |
| AF: Atrial Fibrillation, HR: Hazard Ratio, LA: Left Atrium, NSR: Normal Sinus Rhythm, PAF: Paroxysmal Atrial Fibrillation, RA: Right Atrium, RV: Right Ventricle, SR: Sinus Rhythm | |

| **Supplemental Table S2: Laboratory Findings at Index Hospitalizations.** | |
| --- | --- |
| Lab Value | Median (IQR) |
| Albumin (g/dL) | 3.6 (3.2, 3.9) |
| ALT (unit/L) (N=6655) | 20.0 (14.0, 31.0) |
| AST (unit/L) (N=6558) | 27.0 (20.0, 39.0) |
| BNP (pg/mL) (N=952) | 518.0 (291.0, 878.0) |
| BUN (mg/dL) (N=7707) | 23.0 (16.0, 34.0) |
| Cr (mg/dL) (N=7816) | 1.1 (0.9, 1.6) |
| eGFR (mL/min/1.73 m^2^) (N=7816) | 55.0 (37.0, 74.5) |
| HCO_3_ (mEq/L) (N=7287) | 26.0 (23.0, 29.0) |
| HCT (%/L) (N=7796) | 35.9 (31.3, 40.3) |
| HGB (g/dL) (N=7280) | 11.7 (10.1, 13.2) |
| NTproBNP (pg/mL) (N=5305) | 2391.0 (964.0, 5169.0) |
| Platelets (x10^3^/µL) (N=7800) | 210.0 (161.0, 271.0) |
| Potassium (mEq/L) (N=7213) | 4.2 (3.9, 4.6) |
| Sodium (mEq/L) (N=7811) | 138.0 (135.0, 141.0) |
| Total Protein (g/dL) (N=7033) | 6.6 (6.1, 7.1) |
| WBC (x10^3^/µL) (N=7133) | 8.8 (6.7, 11.8) |

| **Supplemental Table S3: Univariate Cox Analysis** | | | | | | | | |
| --- | --- | --- | --- | --- | --- | --- | --- | --- |
| Feature | Coefficient | HR | HR_Lower_95 | HR_Upper_95 | CI_Width | SE | Z_Score | P_Value |
| Renal Failure | 0.556 | 1.744 | 1.586 | 1.899 | 0.313 | 0.075 | 7.419 | 0.000 |
| Midodrine | 0.514 | 1.672 | 0.946 | 2.584 | 1.639 | 0.398 | 1.292 | 0.196 |
| Chronic Kidney Disease | 0.331 | 1.393 | 1.249 | 1.512 | 0.262 | 0.065 | 5.101 | 0.000 |
| Hypertension | 0.323 | 1.381 | 1.200 | 1.633 | 0.433 | 0.114 | 2.829 | 0.005 |
| COPD | 0.306 | 1.358 | 1.246 | 1.480 | 0.235 | 0.059 | 5.142 | 0.000 |
| Atrial Fibrillation History | 0.304 | 1.355 | 1.241 | 1.493 | 0.251 | 0.063 | 4.851 | 0.000 |
| Atrial Fibrillation on echo | 0.252 | 1.287 | 1.171 | 1.419 | 0.248 | 0.062 | 4.079 | 0.000 |
| Potassium | 0.212 | 1.237 | 1.153 | 1.316 | 0.163 | 0.041 | 5.167 | 0.000 |
| Anxiety | 0.137 | 1.147 | 1.043 | 1.265 | 0.221 | 0.055 | 2.512 | 0.012 |
| Right Ventricular Function | 0.127 | 1.136 | 1.101 | 1.167 | 0.066 | 0.018 | 7.242 | 0.000 |
| Right Ventricular Size | 0.116 | 1.123 | 1.095 | 1.159 | 0.064 | 0.016 | 7.137 | 0.000 |
| Aortic Valve Replacement | 0.113 | 1.119 | 1.072 | 1.169 | 0.096 | 0.024 | 4.639 | 0.000 |
| Depression | 0.095 | 1.099 | 1.005 | 1.222 | 0.217 | 0.053 | 1.784 | 0.074 |
| Mitral Valve Replacement | 0.089 | 1.093 | 1.051 | 1.134 | 0.082 | 0.021 | 4.274 | 0.000 |
| Creatinine | 0.085 | 1.089 | 1.063 | 1.124 | 0.061 | 0.016 | 5.318 | 0.000 |
| Hispanic ethnicity | 0.067 | 1.069 | 1.044 | 1.100 | 0.055 | 0.014 | 4.601 | 0.000 |
| Coronary Artery Disease | 0.060 | 1.062 | 0.935 | 1.187 | 0.252 | 0.058 | 1.027 | 0.304 |
| LV_group | 0.046 | 1.047 | 1.023 | 1.065 | 0.042 | 0.011 | 4.345 | 0.000 |
| Race | 0.044 | 1.045 | 0.982 | 1.126 | 0.144 | 0.036 | 1.218 | 0.223 |
| Age | 0.026 | 1.027 | 1.023 | 1.031 | 0.008 | 0.002 | 13.065 | 0.000 |
| Left Atrial Size | 0.024 | 1.025 | 1.007 | 1.046 | 0.039 | 0.010 | 2.407 | 0.016 |
| Diabetes Mellitus | 0.019 | 1.019 | 0.936 | 1.098 | 0.163 | 0.045 | 0.427 | 0.670 |
| Medial E/e’ Ratio | 0.017 | 1.017 | 1.012 | 1.021 | 0.009 | 0.002 | 6.769 | 0.000 |
| Resp_Rate | 0.016 | 1.016 | 1.008 | 1.024 | 0.016 | 0.004 | 4.012 | 0.000 |
| BUN | 0.013 | 1.013 | 1.012 | 1.016 | 0.004 | 0.001 | 13.124 | 0.000 |
| RVSP | 0.013 | 1.013 | 1.010 | 1.016 | 0.006 | 0.002 | 8.430 | 0.000 |
| WBC | 0.005 | 1.005 | 1.003 | 1.012 | 0.010 | 0.002 | 2.081 | 0.037 |
| Pulse | 0.004 | 1.004 | 1.002 | 1.006 | 0.005 | 0.001 | 3.559 | 0.000 |
| ef | 0.003 | 1.003 | 0.996 | 1.010 | 0.014 | 0.004 | 0.764 | 0.445 |
| ALT | 0.000 | 1.000 | 1.000 | 1.001 | 0.001 | 0.000 | 0.832 | 0.405 |
| AST | 0.000 | 1.000 | 1.000 | 1.001 | 0.001 | 0.000 | 0.830 | 0.407 |
| NTproBNP | 0.000 | 1.000 | 1.000 | 1.000 | 0.000 | 0.000 | 10.311 | 0.000 |
| HCO3 | 0.000 | 1.000 | 0.989 | 1.009 | 0.020 | 0.005 | -0.036 | 0.971 |
| Platelet | -0.001 | 0.999 | 0.999 | 1.000 | 0.001 | 0.000 | -2.036 | 0.042 |
| eGFR | -0.004 | 0.996 | 0.994 | 0.997 | 0.004 | 0.001 | -5.072 | 0.000 |
| O_2__Saturation | -0.006 | 0.994 | 0.985 | 1.004 | 0.020 | 0.005 | -1.314 | 0.189 |
| Hyperlipidemia | -0.007 | 0.993 | 0.901 | 1.100 | 0.199 | 0.053 | -0.135 | 0.893 |
| SBP_echo | -0.010 | 0.990 | 0.987 | 0.992 | 0.005 | 0.001 | -8.721 | 0.000 |
| DBP_echo | -0.012 | 0.988 | 0.985 | 0.991 | 0.006 | 0.002 | -6.954 | 0.000 |
| Gender | -0.025 | 0.975 | 0.906 | 1.070 | 0.164 | 0.045 | -0.555 | 0.579 |
| Rhythm_echo | -0.029 | 0.971 | 0.955 | 0.989 | 0.034 | 0.009 | -3.384 | 0.001 |
| Sodium | -0.030 | 0.971 | 0.963 | 0.978 | 0.016 | 0.004 | -7.401 | 0.000 |
| Body Mass Index | -0.032 | 0.968 | 0.963 | 0.974 | 0.011 | 0.003 | -11.182 | 0.000 |
| Hematocrit | -0.035 | 0.966 | 0.959 | 0.973 | 0.014 | 0.003 | -9.912 | 0.000 |
| Body_temp | -0.036 | 0.964 | 0.903 | 1.020 | 0.117 | 0.033 | -1.098 | 0.272 |
| LVESD | -0.039 | 0.962 | 0.953 | 0.971 | 0.017 | 0.004 | -9.035 | 0.000 |
| LVEDD | -0.040 | 0.961 | 0.955 | 0.967 | 0.012 | 0.003 | -12.123 | 0.000 |
| E-A_ratio | -0.063 | 0.939 | 0.874 | 1.004 | 0.131 | 0.034 | -1.865 | 0.062 |
| HGB | -0.110 | 0.896 | 0.878 | 0.915 | 0.037 | 0.010 | -11.496 | 0.000 |
| Aldo_Antagonist | -0.120 | 0.887 | 0.722 | 1.059 | 0.337 | 0.094 | -1.279 | 0.201 |
| Total protein | -0.283 | 0.753 | 0.710 | 0.797 | 0.087 | 0.022 | -12.703 | 0.000 |
| Albumin | -0.729 | 0.482 | 0.447 | 0.523 | 0.076 | 0.021 | -35.029 | 0.000 |
| SGLT2i | -13.001 | 0.000 | 0.000 | 0.000 | 0.000 | 0.000 | -73927.044 | 0.000 |

| **Supplemental Table S4: Multivariate Cox Analysis** | | | | | | | | |
| --- | --- | --- | --- | --- | --- | --- | --- | --- |
| Feature | Coefficient | HR | HR_Lower_95 | HR_Upper_95 | CI_Width | SE | Z_Score | P_Value |
| Hematocrit | 0.346 | 1.413 | 1.159 | 1.766 | 0.608 | 0.111 | 3.123 | 0.002 |
| Age | 0.328 | 1.388 | 1.301 | 1.493 | 0.193 | 0.037 | 8.931 | 0.000 |
| Renal Failure | 0.175 | 1.192 | 1.132 | 1.262 | 0.130 | 0.028 | 6.288 | 0.000 |
| BUN | 0.172 | 1.188 | 1.125 | 1.266 | 0.141 | 0.031 | 5.512 | 0.000 |
| eGFR | 0.156 | 1.169 | 1.092 | 1.262 | 0.170 | 0.038 | 4.135 | 0.000 |
| LVESD | 0.155 | 1.168 | 0.984 | 1.377 | 0.393 | 0.082 | 1.894 | 0.058 |
| Hispanic ethnicity | 0.111 | 1.117 | 1.064 | 1.176 | 0.112 | 0.026 | 4.319 | 0.000 |
| NTproBNP | 0.102 | 1.108 | 1.049 | 1.173 | 0.125 | 0.028 | 3.644 | 0.000 |
| COPD | 0.089 | 1.093 | 1.041 | 1.147 | 0.106 | 0.026 | 3.483 | 0.000 |
| Depression | 0.089 | 1.093 | 1.030 | 1.148 | 0.118 | 0.027 | 3.272 | 0.001 |
| Right Ventricular Systolic Pressure | 0.088 | 1.092 | 1.023 | 1.150 | 0.128 | 0.028 | 3.148 | 0.002 |
| HCO3 | 0.083 | 1.087 | 1.028 | 1.151 | 0.123 | 0.029 | 2.884 | 0.004 |
| AFIB During_Echo | 0.081 | 1.084 | 0.950 | 1.245 | 0.295 | 0.064 | 1.249 | 0.212 |
| Medial E/e’ Ratio | 0.072 | 1.074 | 1.020 | 1.127 | 0.107 | 0.027 | 2.698 | 0.007 |
| Rhythm During Echo | 0.068 | 1.071 | 0.962 | 1.205 | 0.243 | 0.061 | 1.129 | 0.259 |
| Resp_Rate | 0.068 | 1.070 | 1.026 | 1.125 | 0.099 | 0.023 | 3.011 | 0.003 |
| Right Ventricular Size | 0.068 | 1.070 | 1.006 | 1.147 | 0.140 | 0.033 | 2.066 | 0.039 |
| Ejection Fraction | 0.066 | 1.069 | 0.995 | 1.153 | 0.158 | 0.041 | 1.610 | 0.107 |
| Anxiety | 0.066 | 1.068 | 1.015 | 1.122 | 0.106 | 0.026 | 2.559 | 0.011 |
| Pulse | 0.064 | 1.067 | 1.008 | 1.121 | 0.114 | 0.028 | 2.320 | 0.020 |
| Right Ventricular Function | 0.063 | 1.065 | 0.997 | 1.121 | 0.124 | 0.030 | 2.071 | 0.038 |
| Hypertension | 0.055 | 1.057 | 0.999 | 1.130 | 0.131 | 0.029 | 1.910 | 0.056 |
| Gender | 0.048 | 1.049 | 0.993 | 1.113 | 0.119 | 0.029 | 1.657 | 0.097 |
| Aortic Valve Replacement | 0.042 | 1.043 | 0.995 | 1.088 | 0.093 | 0.024 | 1.767 | 0.077 |
| Total protein | 0.042 | 1.043 | 0.982 | 1.103 | 0.121 | 0.030 | 1.377 | 0.169 |
| Potassium | 0.041 | 1.041 | 0.985 | 1.100 | 0.115 | 0.027 | 1.484 | 0.138 |
| Diabetes Mellitus | 0.037 | 1.037 | 0.987 | 1.087 | 0.099 | 0.025 | 1.468 | 0.142 |
| AST | 0.037 | 1.037 | 0.963 | 1.228 | 0.266 | 0.063 | 0.586 | 0.558 |
| Mitral Valve Replacement | 0.032 | 1.033 | 0.976 | 1.084 | 0.108 | 0.027 | 1.214 | 0.225 |
| Midodrine | 0.024 | 1.024 | 0.974 | 1.065 | 0.091 | 0.022 | 1.062 | 0.288 |
| WBC | 0.022 | 1.022 | 0.986 | 1.067 | 0.082 | 0.021 | 1.049 | 0.294 |
| Diastolic BP-Echo | 0.017 | 1.017 | 0.962 | 1.092 | 0.130 | 0.031 | 0.544 | 0.586 |
| LV_group | 0.012 | 1.012 | 0.959 | 1.069 | 0.109 | 0.027 | 0.429 | 0.668 |
| Chronic Kidney Disease | 0.009 | 1.009 | 0.962 | 1.081 | 0.120 | 0.031 | 0.299 | 0.765 |
| Atrial Fibrillation | 0.009 | 1.009 | 0.952 | 1.069 | 0.117 | 0.030 | 0.311 | 0.756 |
| Coronary Artery Disease | -0.013 | 0.987 | 0.941 | 1.039 | 0.098 | 0.025 | -0.528 | 0.598 |
| O_2__Saturation | -0.015 | 0.985 | 0.936 | 1.039 | 0.103 | 0.027 | -0.560 | 0.576 |
| aldo_antagonist | -0.018 | 0.982 | 0.933 | 1.027 | 0.094 | 0.024 | -0.761 | 0.447 |
| Creatinine | -0.018 | 0.982 | 0.892 | 1.048 | 0.156 | 0.043 | -0.425 | 0.671 |
| Race | -0.020 | 0.981 | 0.938 | 1.041 | 0.103 | 0.027 | -0.721 | 0.471 |
| Left Atrial Size | -0.028 | 0.973 | 0.928 | 1.026 | 0.098 | 0.026 | -1.060 | 0.289 |
| Hyperlipidemia | -0.031 | 0.970 | 0.918 | 1.021 | 0.103 | 0.027 | -1.130 | 0.258 |
| ALT | -0.045 | 0.956 | 0.824 | 1.052 | 0.228 | 0.067 | -0.665 | 0.506 |
| Body_temp | -0.047 | 0.954 | 0.914 | 0.994 | 0.080 | 0.024 | -1.996 | 0.046 |
| E/A Ratio | -0.098 | 0.907 | 0.856 | 0.963 | 0.108 | 0.032 | -3.031 | 0.002 |
| Sodium | -0.112 | 0.894 | 0.847 | 0.944 | 0.097 | 0.028 | -4.064 | 0.000 |
| Platelet | -0.137 | 0.872 | 0.821 | 0.920 | 0.099 | 0.029 | -4.694 | 0.000 |
| Systolic BP-Echo | -0.147 | 0.864 | 0.817 | 0.913 | 0.096 | 0.030 | -4.874 | 0.000 |
| Body Mass Index | -0.171 | 0.843 | 0.778 | 0.901 | 0.123 | 0.037 | -4.683 | 0.000 |
| LVEDD | -0.226 | 0.798 | 0.672 | 0.911 | 0.239 | 0.072 | -3.128 | 0.002 |
| SGLT2 | -0.308 | 0.735 | 0.718 | 0.776 | 0.057 | 0.023 | -13.252 | 0.000 |
| Albumin | -0.360 | 0.698 | 0.661 | 0.737 | 0.076 | 0.030 | -12.037 | 0.000 |
| Hemoglobin | -0.467 | 0.627 | 0.508 | 0.757 | 0.249 | 0.109 | -4.286 | 0.000 |

| **Supplemental Table S5: Binary Classification Model Performance and Comparison** | | |
| --- | --- | --- |
| Model | **XGBoost** | **LightGBM** |
| Model Type | Binary Classification | Binary Classification |
| AUROC_CI | 0.751 (0.727-0.775) | 0.749 (0.721-0.776) |
| AP 95% CI | 0.576 (0.529-0.624) | 0.579 (0.532-0.625) |
| ACCURACY 95% CI | 0.688 (0.666-0.710) | 0.729 (0.707-0.750) |
| BALANCED_ACCURACY 95% CI | 0.687 (0.663-0.713) | 0.632 (0.610-0.656) |
| PRECISION 95% CI | 0.504 (0.465-0.541) | 0.613 (0.557-0.668) |
| RECALL 95% CI | 0.686 (0.646-0.727) | 0.373 (0.331-0.417) |
| F1 95% CI | 0.581 (0.547-0.615) | 0.463 (0.421-0.507) |
| BRIER 95% CI | 0.200 (0.191-0.208) | 0.179 (0.169-0.190) |
| Pairwise (XGBoost vs LightGBM) Model Comparison: | | |
|  | ΔAUC (95% CI) | P value |
| AUROC Difference | 0.002 (-0.032 - 0.040) | < 0.001 |

| **Supplemental Table S6: Survival Models Performance and Comparison** | | | |
| --- | --- | --- | --- |
| Model | Model Type | C-index 95% CI | 12-mo time-dependent AUROC |
| Cox Elastic Net | Survival Analysis | 0.704 (0.680-0.728) | 0.735 (0.692-0.777) |
| RSF | Survival Analysis | 0.711 (0.690-0.734) | 0.750 (0.708-0.789) |
| GBS | Survival Analysis | 0.718 (0.696-0.740) | 0.759 (0.716-0.799) |
| Pairwise Model Comparisons: | | | |
|  | ΔC-Statistic (95% CI) | P value | |
| Cox vs RSF | -0.008 (-0.009 to -0.007) | < 0.001 | |
| Cox vs GBS | -0.014 (-0.015 to -0.013) | < 0.001 | |
| RSF vs GBS | -0.006 (-0.007 to -0.005) | < 0.001 | |

| **Supplemental Table S7: Feature-consistency table based on SHAP summary statistics for each model** | | | | | | |
| --- | --- | --- | --- | --- | --- | --- |
|  | XGBoost | LightGBM | Cox Elastic Net | RSF | GBS | Mean across Models |
| Albumin | 90.14 | 90.35 | 100.00 | 100.00 | 100.00 | 96.10 |
| Age | 100.00 | 100.00 | 95.59 | 83.69 | 99.61 | 95.78 |
| BUN | 65.18 | 64.05 | 41.35 | 81.78 | 91.54 | 68.78 |
| Renal Failure | 64.80 | 63.81 | 65.60 | 43.08 | 66.13 | 60.68 |
| NTproBNP | 56.74 | 48.99 | 24.28 | 78.43 | 46.10 | 50.91 |
| Systolic BP-Echo | 46.79 | 44.51 | 38.13 | 59.42 | 55.39 | 48.85 |
| Body Mass Index | 42.33 | 42.46 | 42.56 | 54.81 | 52.61 | 46.95 |
| Hemoglobin | 45.46 | 41.59 | 36.65 | 41.45 | 48.20 | 42.67 |
| COPD | 51.60 | 51.45 | 35.34 | 24.67 | 47.97 | 42.21 |
| Sodium | 43.63 | 38.50 | 21.87 | 40.05 | 58.38 | 40.49 |
| eGFR | 39.55 | 28.68 | 37.85 | 51.42 | 43.17 | 40.13 |
| LVEDD | 30.17 | 26.02 | 30.77 | 43.82 | 39.58 | 34.07 |
| Platelet | 36.60 | 30.43 | 26.30 | 29.09 | 34.86 | 31.46 |
| Right Ventricular Systolic Pressure | 26.41 | 26.33 | 23.38 | 38.61 | 41.72 | 31.29 |
| Pulse | 35.46 | 33.67 | 21.79 | 23.84 | 39.53 | 30.86 |
| HCO3 | 25.24 | 24.75 | 24.80 | 30.74 | 46.13 | 30.33 |
| ALT | 33.66 | 31.75 | 0.00 | 23.38 | 55.70 | 28.90 |
| AST | 31.33 | 31.12 | 0.00 | 28.89 | 49.00 | 28.07 |
| Right Ventricular Size | 18.75 | 22.22 | 22.52 | 34.08 | 36.89 | 26.89 |
| Resp Rate | 19.23 | 17.57 | 15.78 | 26.60 | 39.68 | 23.77 |
| WBC | 20.69 | 18.83 | 2.74 | 30.02 | 45.10 | 23.48 |
| Depression | 25.61 | 24.92 | 28.20 | 6.40 | 17.10 | 20.45 |
| Potassium | 19.91 | 17.20 | 14.21 | 21.16 | 29.67 | 20.43 |
| Creatinine | 8.96 | 6.22 | 0.00 | 40.76 | 44.04 | 20.00 |
| Total protein | 14.62 | 12.86 | 4.57 | 32.75 | 28.96 | 18.75 |
| HCT | 10.81 | 13.34 | 0.00 | 33.43 | 34.12 | 18.34 |
| Right Ventricular Function | 7.14 | 8.82 | 16.14 | 30.94 | 26.85 | 17.98 |
| Anxiety | 17.91 | 16.93 | 19.42 | 8.42 | 19.73 | 16.48 |
| Body_temp | 13.50 | 12.40 | 8.13 | 15.38 | 31.26 | 16.13 |
| Medial E/e’ Ratio | 9.24 | 11.47 | 9.56 | 21.83 | 23.41 | 15.10 |
| O2_sat | 11.74 | 13.25 | 2.40 | 20.08 | 26.49 | 14.79 |
| Diastolic BP-Echo | 10.16 | 13.01 | 0.00 | 20.82 | 23.51 | 13.50 |
| LVESD | 6.88 | 7.06 | 0.00 | 24.92 | 20.38 | 11.85 |
| AVR_gp | 9.30 | 8.43 | 10.42 | 11.97 | 15.43 | 11.11 |
| Atrial Fibrillation | 12.41 | 10.55 | 0.00 | 11.64 | 14.45 | 9.81 |
| Hypertension | 14.05 | 18.53 | 8.17 | 2.51 | 5.62 | 9.77 |
| Diabetes Mellitus | 10.49 | 10.01 | 8.57 | 5.96 | 10.29 | 9.06 |
| Ejection Fraction | 6.00 | 2.80 | 0.00 | 14.94 | 16.60 | 8.07 |
| Gender | 6.60 | 6.01 | 7.92 | 6.14 | 11.79 | 7.69 |
| Rhythm during Echo | 4.18 | 2.23 | 0.00 | 12.71 | 15.18 | 6.86 |
| Mitral Valve Replacement | 3.38 | 3.51 | 1.52 | 9.62 | 14.01 | 6.41 |
| Hispanic ethnicity | 2.12 | 4.01 | 16.05 | 2.54 | 5.51 | 6.05 |
| Left atrial Size | 1.78 | 1.85 | 3.54 | 10.49 | 10.46 | 5.62 |
| Chronic Kidney Disease | 3.46 | 0.22 | 0.14 | 11.34 | 8.73 | 4.78 |
| Hyperlipidemia | 3.69 | 2.27 | 4.32 | 5.61 | 5.17 | 4.21 |
| Atrial Fibrillation During Echo | 0.00 | 0.00 | 4.89 | 6.30 | 3.19 | 2.87 |
| CAD | 0.19 | 0.00 | 0.00 | 4.66 | 4.84 | 1.94 |
| Aldosterone Antagonist | 0.00 | 0.11 | 1.11 | 0.26 | 0.95 | 0.49 |
| Midodrine | 0.00 | 0.00 | 1.31 | 0.05 | 0.28 | 0.33 |
| Race | 0.13 | 0.00 | 0.00 | 0.59 | 0.66 | 0.28 |
| SGLT2i | 0.00 | 0.00 | 0.00 | 0.00 | 0.00 | 0.00 |

| **Supplemental Table S8: Binary prediction LIME summary table** | | | | |
| --- | --- | --- | --- | --- |
| model | case | feature | weight | predicted probability |
| XGBoost | High Risk | age > 86.00 | 0.149 | 0.941 |
| XGBoost | High Risk | albumin <= 3.20 | 0.138 | 0.941 |
| XGBoost | High Risk | BUN > 34.00 | 0.079 | 0.941 |
| XGBoost | High Risk | NTproBNP > 5873.50 | 0.077 | 0.941 |
| XGBoost | High Risk | 0.00 < renal failure <= 1.00 | 0.075 | 0.941 |
| XGBoost | High Risk | 0.00 < COPD <= 1.00 | 0.069 | 0.941 |
| XGBoost | High Risk | BMI <= 25.30 | 0.058 | 0.941 |
| XGBoost | High Risk | SBP_echo <= 114.00 | 0.057 | 0.941 |
| XGBoost | High Risk | ALT <= 15.00 | 0.053 | 0.941 |
| XGBoost | High Risk | LVEDD <= 42.00 | 0.052 | 0.941 |
| XGBoost | High Risk | Platelet > 271.00 | -0.038 | 0.941 |
| XGBoost | High Risk | Resp_rate > 22.00 | 0.038 | 0.941 |
| XGBoost | High Risk | 37.19 < eGFR <= 55.01 | -0.038 | 0.941 |
| XGBoost | High Risk | 10.10 < HGB <= 11.70 | 0.034 | 0.941 |
| XGBoost | High Risk | SGLT2 <= 0.00 | 0.017 | 0.941 |
| XGBoost | Median Risk | albumin <= 3.20 | 0.129 | 0.454 |
| XGBoost | Median Risk | eGFR > 74.23 | 0.069 | 0.454 |
| XGBoost | Median Risk | 0.00 < renal failure <= 1.00 | 0.069 | 0.454 |
| XGBoost | Median Risk | BUN <= 16.00 | -0.069 | 0.454 |
| XGBoost | Median Risk | Sodium <= 135.00 | 0.068 | 0.454 |
| XGBoost | Median Risk | COPD <= 0.00 | -0.065 | 0.454 |
| XGBoost | Median Risk | BMI <= 25.30 | 0.056 | 0.454 |
| XGBoost | Median Risk | SBP_echo <= 114.00 | 0.049 | 0.454 |
| XGBoost | Median Risk | NTproBNP <= 1298.70 | -0.044 | 0.454 |
| XGBoost | Median Risk | Pulse <= 70.25 | -0.044 | 0.454 |
| XGBoost | Median Risk | 11.70 < HGB <= 13.20 | -0.043 | 0.454 |
| XGBoost | Median Risk | LVEDD <= 42.00 | 0.040 | 0.454 |
| XGBoost | Median Risk | midodrine <= 0.00 | 0.033 | 0.454 |
| XGBoost | Median Risk | WBC > 11.60 | 0.030 | 0.454 |
| XGBoost | Median Risk | SGLT2 <= 0.00 | -0.024 | 0.454 |
| XGBoost | Low Risk | age <= 68.00 | -0.150 | 0.026 |
| XGBoost | Low Risk | albumin > 3.90 | -0.122 | 0.026 |
| XGBoost | Low Risk | renal failure <= 0.00 | -0.074 | 0.026 |
| XGBoost | Low Risk | BUN <= 16.00 | -0.071 | 0.026 |
| XGBoost | Low Risk | eGFR > 74.23 | 0.071 | 0.026 |
| XGBoost | Low Risk | HGB > 13.20 | -0.067 | 0.026 |
| XGBoost | Low Risk | COPD <= 0.00 | -0.064 | 0.026 |
| XGBoost | Low Risk | SGLT2 <= 0.00 | 0.052 | 0.026 |
| XGBoost | Low Risk | 21.00 < AST <= 27.90 | -0.043 | 0.026 |
| XGBoost | Low Risk | 138.00 < Sodium <= 141.00 | -0.039 | 0.026 |
| XGBoost | Low Risk | NTproBNP <= 1298.70 | -0.039 | 0.026 |
| XGBoost | Low Risk | depression <= 0.00 | -0.039 | 0.026 |
| XGBoost | Low Risk | Pulse <= 70.25 | -0.038 | 0.026 |
| XGBoost | Low Risk | Platelet > 271.00 | -0.031 | 0.026 |
| XGBoost | Low Risk | RVSP <= 36.00 | -0.029 | 0.026 |
| LightGBM | High Risk | 0.00 < renal failure <= 1.00 | 0.074 | 0.880 |
| LightGBM | High Risk | NTproBNP > 5873.50 | 0.066 | 0.880 |
| LightGBM | High Risk | SGLT2 <= 0.00 | -0.062 | 0.880 |
| LightGBM | High Risk | ALT <= 15.00 | 0.057 | 0.880 |
| LightGBM | High Risk | SBP_echo <= 114.00 | 0.057 | 0.880 |
| LightGBM | High Risk | Platelet <= 161.00 | 0.049 | 0.880 |
| LightGBM | High Risk | 0.00 < COPD <= 1.00 | 0.048 | 0.880 |
| LightGBM | High Risk | HGB <= 10.10 | 0.037 | 0.880 |
| LightGBM | High Risk | 3.60 < albumin <= 3.90 | -0.034 | 0.880 |
| LightGBM | High Risk | 138.00 < Sodium <= 141.00 | -0.031 | 0.880 |
| LightGBM | High Risk | 23.00 < BUN <= 34.00 | 0.029 | 0.880 |
| LightGBM | High Risk | 0.00 < anxiety <= 1.00 | 0.029 | 0.880 |
| LightGBM | High Risk | WBC > 11.60 | 0.028 | 0.880 |
| LightGBM | High Risk | Body_temp > 36.90 | -0.026 | 0.880 |
| LightGBM | High Risk | HCO3 <= 23.00 | -0.026 | 0.880 |
| LightGBM | Median Risk | albumin <= 3.20 | 0.139 | 0.277 |
| LightGBM | Median Risk | AST > 39.00 | 0.073 | 0.277 |
| LightGBM | Median Risk | renal failure <= 0.00 | -0.062 | 0.277 |
| LightGBM | Median Risk | BUN <= 16.00 | -0.060 | 0.277 |
| LightGBM | Median Risk | ALT > 32.00 | -0.053 | 0.277 |
| LightGBM | Median Risk | COPD <= 0.00 | -0.047 | 0.277 |
| LightGBM | Median Risk | Platelet <= 161.00 | 0.044 | 0.277 |
| LightGBM | Median Risk | Pulse <= 70.25 | -0.037 | 0.277 |
| LightGBM | Median Risk | depression <= 0.00 | -0.034 | 0.277 |
| LightGBM | Median Risk | HGB <= 10.10 | 0.033 | 0.277 |
| LightGBM | Median Risk | LVEDD > 51.00 | -0.032 | 0.277 |
| LightGBM | Median Risk | RV_size_gp <= 1.00 | -0.031 | 0.277 |
| LightGBM | Median Risk | Body_temp <= 36.45 | 0.030 | 0.277 |
| LightGBM | Median Risk | RVSP > 55.00 | 0.028 | 0.277 |
| LightGBM | Median Risk | SGLT2 <= 0.00 | 0.021 | 0.277 |
| LightGBM | Low Risk | age <= 68.00 | -0.135 | 0.013 |
| LightGBM | Low Risk | renal failure <= 0.00 | -0.059 | 0.013 |
| LightGBM | Low Risk | BMI > 36.85 | -0.058 | 0.013 |
| LightGBM | Low Risk | BUN <= 16.00 | -0.056 | 0.013 |
| LightGBM | Low Risk | COPD <= 0.00 | -0.052 | 0.013 |
| LightGBM | Low Risk | eGFR > 74.23 | 0.050 | 0.013 |
| LightGBM | Low Risk | HGB > 13.20 | -0.045 | 0.013 |
| LightGBM | Low Risk | SGLT2 <= 0.00 | 0.042 | 0.013 |
| LightGBM | Low Risk | 21.00 < AST <= 27.90 | -0.041 | 0.013 |
| LightGBM | Low Risk | LVEDD > 51.00 | -0.032 | 0.013 |
| LightGBM | Low Risk | 128.00 < SBP_echo <= 144.00 | -0.032 | 0.013 |
| LightGBM | Low Risk | 138.00 < Sodium <= 141.00 | -0.032 | 0.013 |
| LightGBM | Low Risk | RV_size_gp <= 1.00 | -0.029 | 0.013 |
| LightGBM | Low Risk | RVSP <= 36.00 | -0.026 | 0.013 |
| LightGBM | Low Risk | 211.00 < Platelet <= 271.00 | -0.025 | 0.013 |

| **Supplemental Table S9: Variable value missingness** | | |
| --- | --- | --- |
| Variable | Missing Count | Missing Percentage |
| LOS | 0 | 0.00 |
| albumin | 0 | 0.00 |
| d_albumin | 0 | 0.00 |
| albumin_time | 0 | 0.00 |
| EF | 0 | 0.00 |
| Gender | 0 | 0.00 |
| age | 0 | 0.00 |
| HTN | 0 | 0.00 |
| DM | 0 | 0.00 |
| Hyperlipidemia | 0 | 0.00 |
| CKD | 0 | 0.00 |
| COPD | 0 | 0.00 |
| CAD | 0 | 0.00 |
| anxiety | 0 | 0.00 |
| depression | 0 | 0.00 |
| pulmonary_fibrosis | 0 | 0.00 |
| renal_failure | 0 | 0.00 |
| race_gp | 0 | 0.00 |
| hispanic_ethnicity | 0 | 0.00 |
| TIA_stroke | 0 | 0.00 |
| tm_dth1 | 0 | 0.00 |
| ev_dth1 | 0 | 0.00 |
| male | 0 | 0.00 |
| age_80 | 0 | 0.00 |
| no_htn | 0 | 0.00 |
| alb_32 | 0 | 0.00 |
| score5 | 0 | 0.00 |
| score4 | 0 | 0.00 |
| yr_admit | 0 | 0.00 |
| BMI | 16 | 0.20 |
| creatinine | 24 | 0.31 |
| eGFR | 24 | 0.31 |
| wt_echo | 26 | 0.33 |
| Sodium | 29 | 0.37 |
| ht_echo | 33 | 0.42 |
| bmi_echo | 34 | 0.43 |
| Platelet | 40 | 0.51 |
| HCT | 44 | 0.56 |
| BUN | 133 | 1.70 |
| HR_echo | 159 | 2.03 |
| RV_size_gp | 191 | 2.44 |
| SBP_echo | 199 | 2.54 |
| DBP_echo | 204 | 2.60 |
| RV_function_gp | 298 | 3.80 |
| SGLT2 | 503 | 6.42 |
| aldo_antagonist | 503 | 6.42 |
| midodrine | 503 | 6.42 |
| LVEDD | 508 | 6.48 |
| Rhythm_echo | 529 | 6.75 |
| AFIB_echo | 529 | 6.75 |
| HCO3 | 553 | 7.05 |
| HGB | 560 | 7.14 |
| MVR_gp | 593 | 7.56 |
| K | 627 | 8.00 |
| WBC | 707 | 9.02 |
| Total_protein | 807 | 10.29 |
| LVESD | 809 | 10.32 |
| LA_size_gp | 830 | 10.59 |
| RA_size_gp | 842 | 10.74 |
| O2_sat | 931 | 11.88 |
| ALT | 1185 | 15.11 |
| AST | 1282 | 16.35 |
| AVR_gp | 1362 | 17.37 |
| med_E_e_prime | 1498 | 19.11 |
| e_ep_20 | 1498 | 19.11 |
| score4_comp | 1498 | 19.11 |
| Resp_rate | 1557 | 19.86 |
| RVSP | 1640 | 20.92 |
| Weight | 2025 | 25.83 |
| Height | 2228 | 28.42 |
| Pulse | 2302 | 29.36 |
| Body_temp | 2525 | 32.21 |
| NTproBNP | 2535 | 32.33 |
| ntprobnp_5000 | 2535 | 32.33 |
| LV_group | 2838 | 36.20 |
| E_A_ratio | 3151 | 40.19 |
| score5_comp | 3504 | 44.69 |
| lat_E_e_prime | 3670 | 46.81 |
| ICD10_death | 6407 | 81.72 |
| cardiac_death | 6407 | 81.72 |
| AS_gp | 6493 | 82.82 |
| BNP | 6888 | 87.86 |

**SUPPLEMENTAL FIGURE LEGENDS**

**Supplemental Figure S1:** **Calibration curves (binary).**
**Caption:** Calibration plots comparing predicted mortality probabilities to observed event fractions for XGBoost (blue) and LightGBM (green) on the test set. The dashed diagonal line represents perfect calibration. Both models demonstrated acceptable calibration with mild under-prediction at very low risk strata. Predicted probabilities were binned into deciles; markers represent bin means. Overall, predictions closely followed the ideal calibration line, supporting model reliability for clinical risk estimation.

**Supplemental Figure S2:** **Calibration of survival models at 12 months.
Caption:** Predicted 12-month event probabilities (x-axis) are plotted against Kaplan-Meier-observed event probabilities (y-axis) across deciles of predicted risk for Cox Elastic Net, Random Survival Forest (RSF), and Gradient Boosting Survival (GBS) models. Each point represents one decile group of the held-out test set (n=1,567). The dashed diagonal line represents perfect calibration. Mean calibration error (MCE), defined as the mean absolute difference between predicted and observed probabilities across deciles, was 0.014 for Cox Elastic Net, 0.020 for GBS, and 0.024 for RSF. All three models demonstrated good calibration within the observed risk range (0–35%), consistent with the overall 12-month event rate of 31.5%.

**Supplemental Figure S3**: **Top protective and risk factors.**
**Caption:** Four-panel summary of Cox regression results. Upper panels display the top 15 protective factors (HR<1.0) from univariate (left) and multivariable (right) analyses. Lower panels display the top 15 risk factors (HR>1.0). Dark bars indicate statistical significance (p<0.05). Albumin, hemoglobin, and SGLT2 inhibitors were consistently protective; age, renal failure, and hematocrit were consistent risk factors across both analytical approaches. HR, hazard ratio; SGLT2, sodium-glucose cotransporter-2.

**Supplemental Figure S4:** **Per-model SHAP summaries.**
**Caption:** Horizontal bar plots showing normalized mean absolute SHAP values (0–100 scale) for binary classifiers (left: XGBoost, LightGBM) and survival models (right: Cox, RSF, GBS). Feature rankings were highly consistent across model families, with albumin, age, BUN, and renal failure ranking highest. This cross-method agreement supports the robustness of identified predictors. SHAP, SHapley Additive exPlanations; RSF, Random Survival Forest; GBS, Gradient Boosting Survival; BUN, blood urea nitrogen.

**Supplemental Figure S5:** **Radar plot of top-8 features across the five models.**
**Caption:** Radar plot displaying normalized SHAP importance (0–100 scale) for the eight most influential predictors across all five models. Each colored polygon represents one model: XGBoost (teal), LightGBM (green), Cox Elastic Net (coral), RSF (purple), and GBS (gold). Albumin and age approached maximum importance across all methods. BUN showed highest relative weight in GBS; NTproBNP in RSF. The overlapping polygons demonstrate cross-model consistency. BUN, blood urea nitrogen; NTproBNP, N-terminal pro-B-type natriuretic peptide; HGB, hemoglobin; BMI, body mass index; SBP_echo, systolic blood pressure at echocardiography.

**Supplemental Figure S6:** **Inter-model importance correlation heat-map.**
**Caption:** Pairwise Pearson correlation matrix of SHAP-derived feature importance rankings across all five models. Color intensity reflects correlation strength (scale: 0–1). Binary classifiers showed near-perfect agreement (XGBoost–LightGBM: r=0.994). Survival tree-based models also correlated strongly (RSF–GBS: r=0.913). The lowest correlation (Cox–RSF: r=0.775) remained substantial, indicating that all modeling approaches identified similar predictive signals despite differing algorithmic structures. SHAP, SHapley Additive exPlanations; RSF, Random Survival Forest; GBS, Gradient Boosting Survival.

**Supplemental Figure S7:** **Cross-model normalized comparisons.**
**Caption:** Left panel: Horizontal bar plot of normalized mean SHAP values for the top-20 features, with color-coded contributions from each model. Right panel: Heatmap displaying normalized SHAP importance for the top-25 features (rows) across all five models (columns). Warmer colors indicate higher importance. Albumin, age, BUN, and renal failure consistently ranked highest across methods, while lower-ranked features showed greater model-specific variability. SHAP, SHapley Additive exPlanations; BUN, blood urea nitrogen; NTproBNP, N-terminal pro-B-type natriuretic peptide; eGFR, estimated glomerular filtration rate.

**Supplemental Figure S8:** **XGBoost global (SHAP) explainability.**
**Caption:** Beeswarm plot displaying global feature importance and directional effects for the XGBoost classifier. Each dot represents one patient; horizontal position indicates SHAP value (impact on mortality prediction), and color represents feature value (red=high, blue=low). Features are ranked by mean absolute SHAP value. Older age and higher BUN pushed predictions toward mortality; higher albumin and hemoglobin were protective. SHAP, SHapley Additive exPlanations; BUN, blood urea nitrogen; NTproBNP, N-terminal pro-B-type natriuretic peptide; HGB, hemoglobin; eGFR, estimated glomerular filtration rate; LVEDD, left ventricular end-diastolic dimension; RVSP, right ventricular systolic pressure.

**Supplemental Figure S9:** **LightGBM global (SHAP) explainability.**
**Caption:** Beeswarm plot of global SHAP values for the LightGBM classifier. Each dot represents one patient, with horizontal position showing SHAP impact and color indicating feature value (red=high, blue=low). Feature rankings closely paralleled XGBoost, with age, albumin, BUN, and renal failure as top predictors. Higher albumin and hemoglobin values (blue, leftward) reduced predicted mortality risk. SHAP, SHapley Additive exPlanations; BUN, blood urea nitrogen; NTproBNP, N-terminal pro-B-type natriuretic peptide; HGB, hemoglobin; SBP_echo, systolic blood pressure at echocardiography; eGFR, estimated glomerular filtration rate.

**Supplemental Figure S10:** **Cox Elastic Net global (SHAP) explainability.**
**Caption:** Beeswarm plot of SHAP values for the regularized Cox proportional hazards model. Albumin ranked highest; low values (blue) produced positive SHAP contributions, indicating increased hazard. Age and renal failure followed, with older age and renal impairment associated with higher risk. The vertical spread reflects penalized coefficient constraints. Color gradient: red=high feature value, blue=low. SHAP, SHapley Additive exPlanations; BUN, blood urea nitrogen; eGFR, estimated glomerular filtration rate; HGB, hemoglobin; LVEDD, left ventricular end-diastolic dimension; RVSP, right ventricular systolic pressure; RV, right ventricular.

**Supplemental Figure S11: RSF global (SHAP) explainability.**
**Caption:** Beeswarm plot of SHAP values for the Random Survival Forest (RSF) model. Albumin, age, BUN, and NTproBNP were the strongest contributors. Low albumin (blue dots, rightward) and high NTproBNP (red dots, rightward) increased predicted risk. RSF placed relatively higher importance on NTproBNP compared to other models. Color gradient: red=high feature value, blue=low. SHAP, SHapley Additive exPlanations; BUN, blood urea nitrogen; NTproBNP, N-terminal pro-B-type natriuretic peptide; eGFR, estimated glomerular filtration rate; HGB, hemoglobin; HCT, hematocrit; RVSP, right ventricular systolic pressure.

**Supplemental Figure S12:** **GBS global (SHAP) explainability.**
**Caption:** Beeswarm plot of SHAP values for the Gradient Boosting Survival (GBS) model. Albumin and age ranked highest, followed by BUN and renal failure. Low albumin values (blue) and older age (red) pushed predictions toward higher mortality risk. GBS showed the highest relative importance for BUN among survival models. Color gradient: red=high feature value, blue=low. SHAP, SHapley Additive exPlanations; BUN, blood urea nitrogen; HGB, hemoglobin; NTproBNP, N-terminal pro-B-type natriuretic peptide; eGFR, estimated glomerular filtration rate; LVEDD, left ventricular end-diastolic dimension.

**Supplemental Figure S13:** **XGBoost model LIME explanations (local, or patient-level).**
**Caption:** Local Interpretable Model-agnostic Explanations (LIME) for representative XGBoost predictions across risk strata: high-risk (predicted probability=0.941), median-risk (0.454), and low-risk (0.026). Green bars indicate features reducing predicted risk; red bars indicate features increasing risk. High-risk profiles showed very old age, low albumin, elevated BUN, and high NTproBNP. Low-risk profiles featured preserved renal function, higher albumin, and SGLT2 inhibitor use. Feature conditions are listed on the y-axis. BUN, blood urea nitrogen; NTproBNP, N-terminal pro-B-type natriuretic peptide; HGB, hemoglobin; eGFR, estimated glomerular filtration rate.

**Supplemental Figure S14:** **LightGBM model LIME explanations (local, or patient-level).
Caption:** LIME explanations for representative LightGBM predictions: high-risk (predicted probability=0.880), median-risk (0.277), and low-risk (0.013). Green bars represent protective contributions; red bars represent risk-increasing contributions. The high-risk patient exhibited renal failure, markedly elevated NTproBNP, and absence of SGLT2 therapy. The low-risk patient showed younger age, preserved renal function, higher hemoglobin, and SGLT2 inhibitor use. Patterns mirrored XGBoost explanations, supporting cross-model consistency. LIME, Local Interpretable Model-agnostic Explanations; NTproBNP, N-terminal pro-B-type natriuretic peptide; HGB, hemoglobin; eGFR, estimated glomerular filtration rate.

**Supplemental Figure S15:** **Cox Elastic Net model LIME explanations (local, or patient-level).**
**Caption:** SHAP-based waterfall explanations for representative Cox Elastic Net predictions across risk strata: high-risk (risk score=3.036), median-risk (0.016), and low-risk (−2.616). Green bars indicate protective feature contributions (reducing hazard); red bars indicate risk-increasing contributions. High-risk profiles were characterized by low eGFR, advanced age, and low albumin. Low-risk profiles showed younger age, higher albumin, and preserved renal function. SHAP, SHapley Additive exPlanations; eGFR, estimated glomerular filtration rate; LVEDD, left ventricular end-diastolic dimension; BUN, blood urea nitrogen; RVSP, right ventricular systolic pressure.

**Supplemental Figure S16:** **RSF model LIME explanations (local, or patient-level).
Caption:** SHAP-based explanations for representative Random Survival Forest (RSF) predictions: high-risk (risk score=413.783), median-risk (114.698), and low-risk (5.642). Green bars indicate features increasing predicted risk; red bars indicate protective contributions (note: direction reflects survival model output). High-risk profiles showed low SBP_echo, elevated NTproBNP, and larger LVEDD. Low-risk profiles demonstrated lower NTproBNP, preserved renal function, and higher hemoglobin. SHAP, SHapley Additive exPlanations; SBP_echo, systolic blood pressure at echocardiography; NTproBNP, N-terminal pro-B-type natriuretic peptide; LVEDD, left ventricular end-diastolic dimension; eGFR, estimated glomerular filtration rate; BUN, blood urea nitrogen.

**Supplemental Figure S17:** **GBS model LIME explanations (local, or patient-level).**
**Caption:** SHAP-based explanations for representative Gradient Boosting Survival (GBS) predictions: high-risk (risk score=3.587), median-risk (−0.044), and low-risk (−2.448). Green bars indicate features increasing risk; red bars indicate protective contributions. The high-risk patient exhibited low albumin, low platelet count, and hyponatremia. The low-risk patient showed higher albumin, hemoglobin, and sodium with lower NTproBNP. These patient-level narratives align with global SHAP findings, confirming model interpretability. SHAP, SHapley Additive exPlanations; NTproBNP, N-terminal pro-B-type natriuretic peptide; HGB, hemoglobin; eGFR, estimated glomerular filtration rate; HCT, hematocrit.

**Figure S1: Calibration curves (binary).**


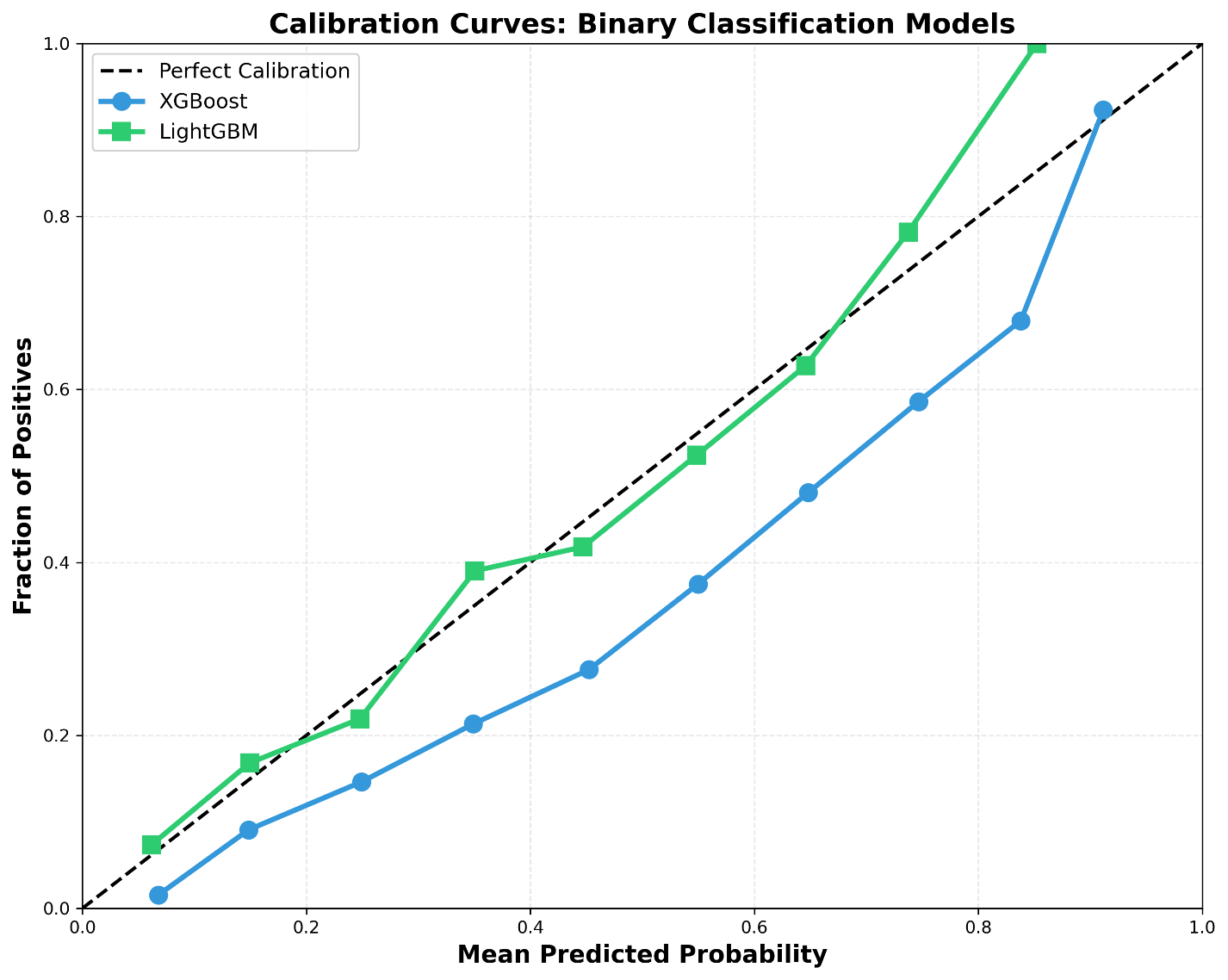


**Figure S2: Twelve-month calibration of survival models.**


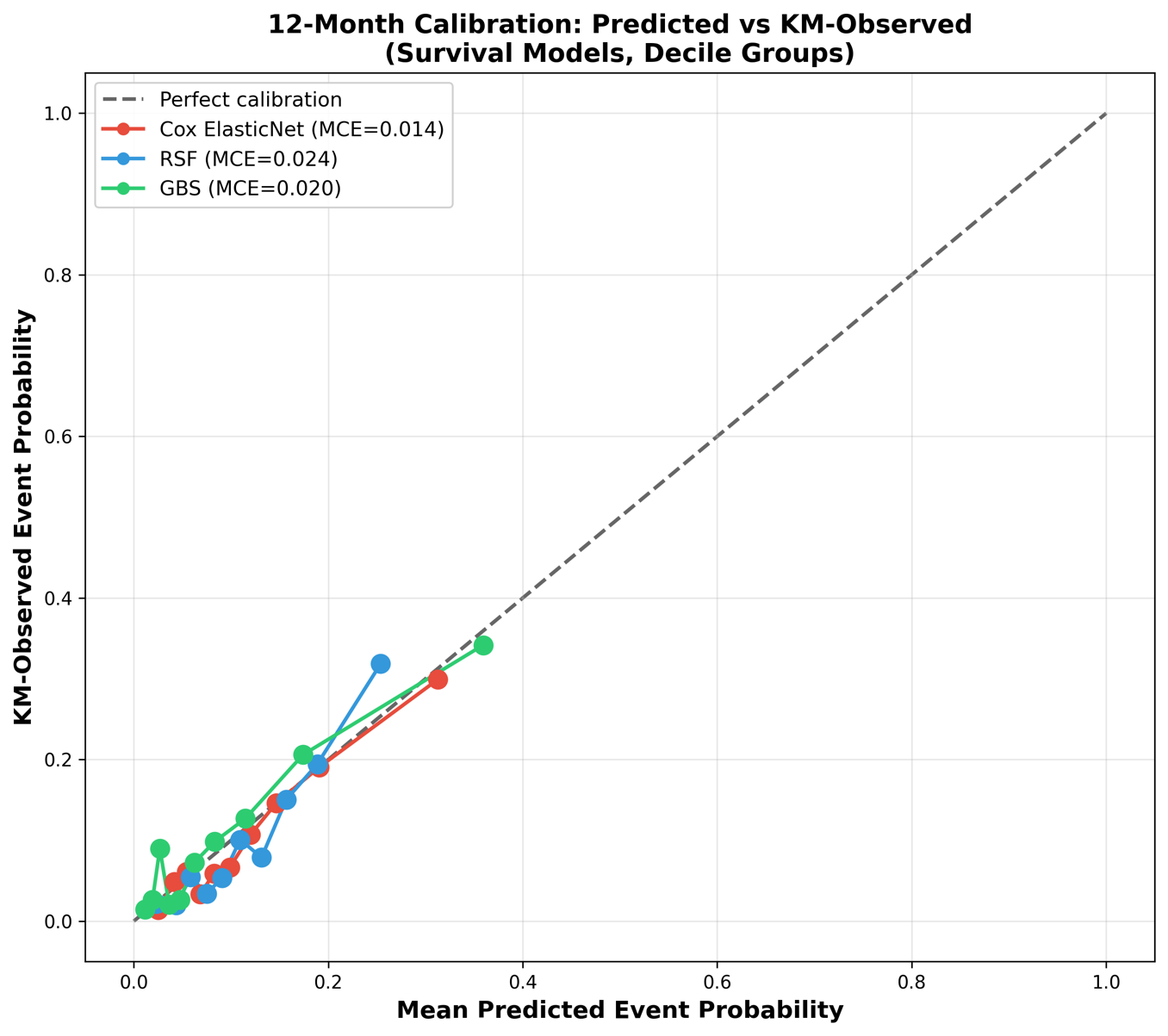


**Figure S3: Top protective and risk factors.**


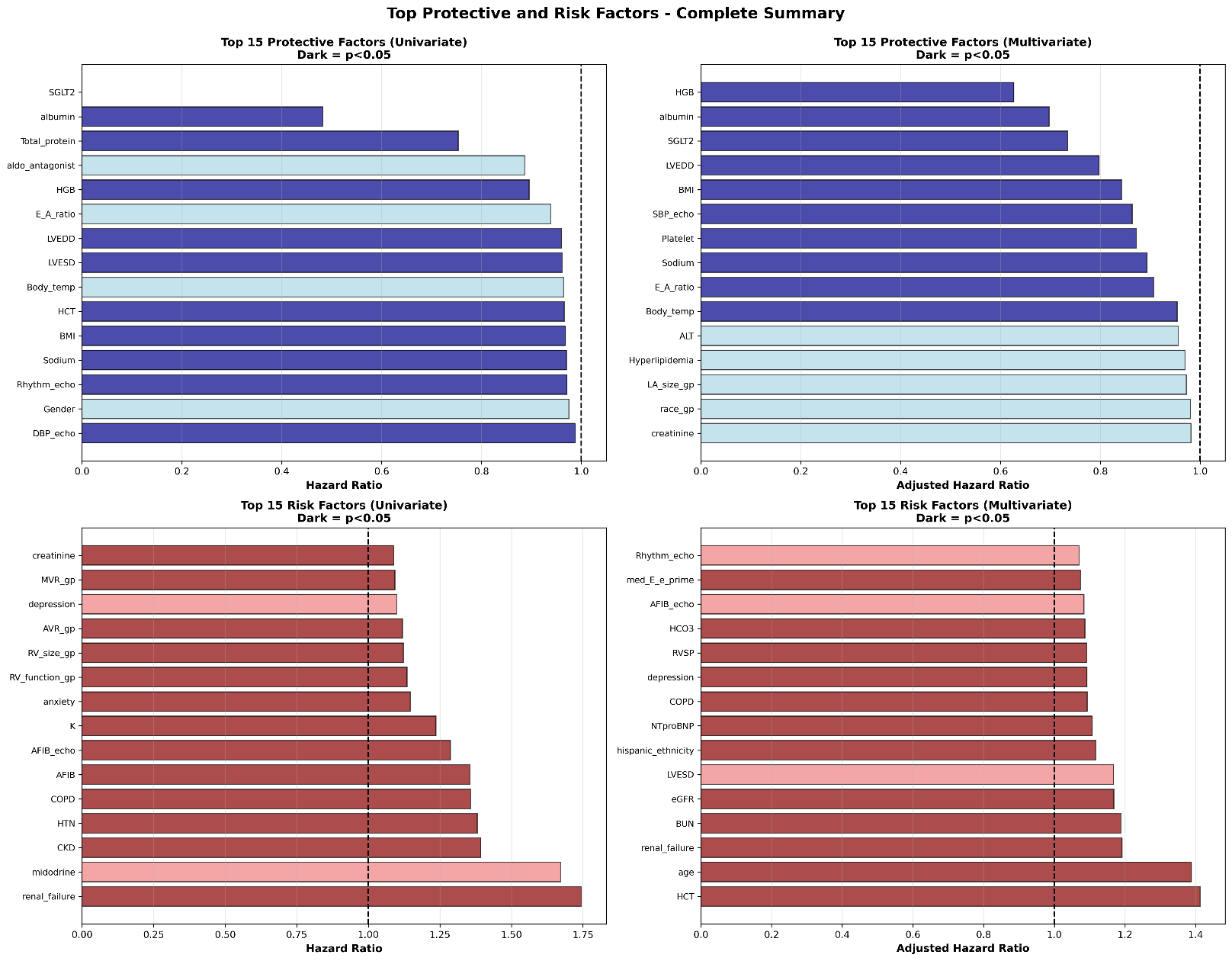


**Figure S4: Per-model SHAP summaries.**


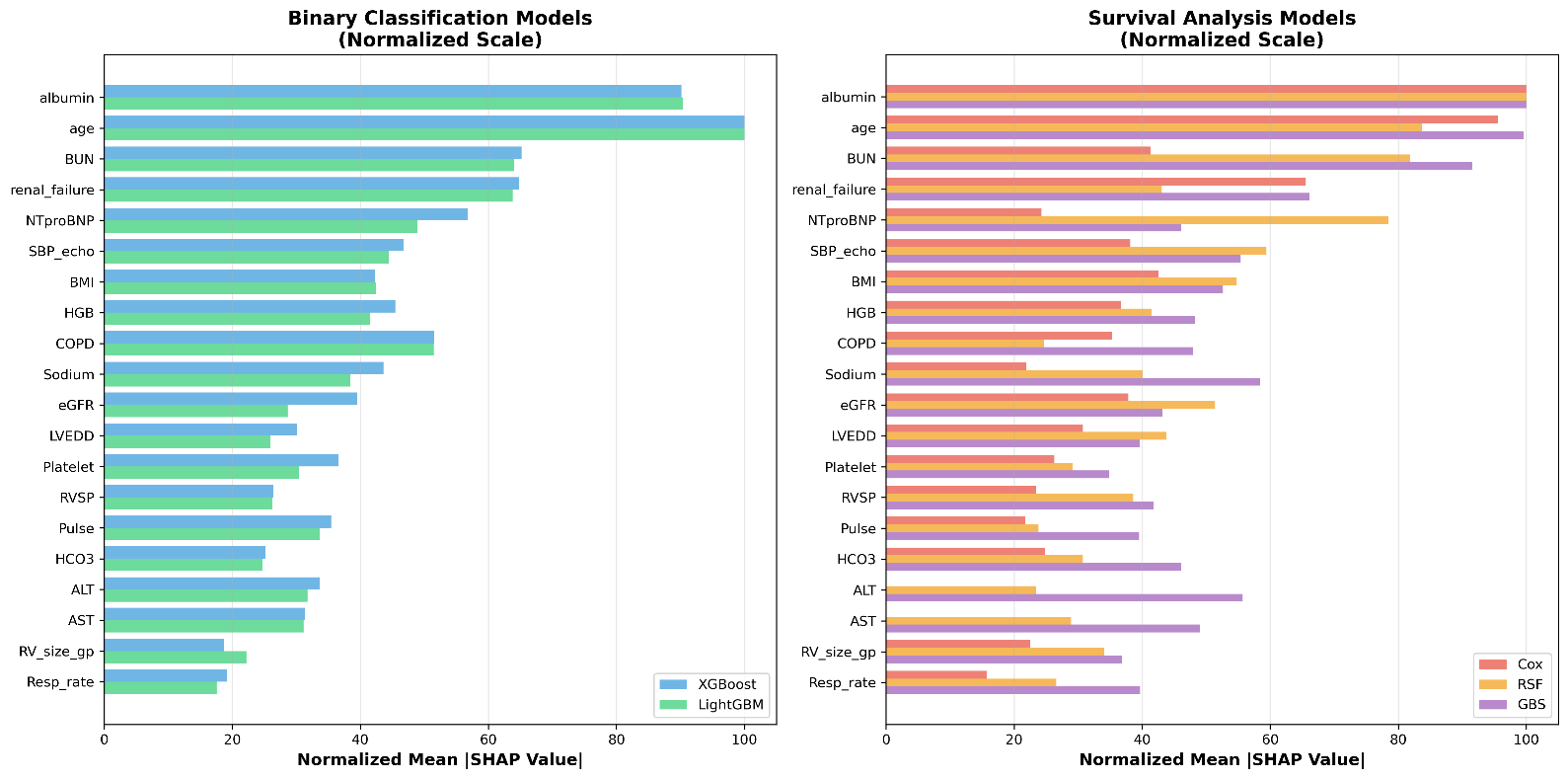


**Figure S5: Radar plot of top-8 features across the five models.**


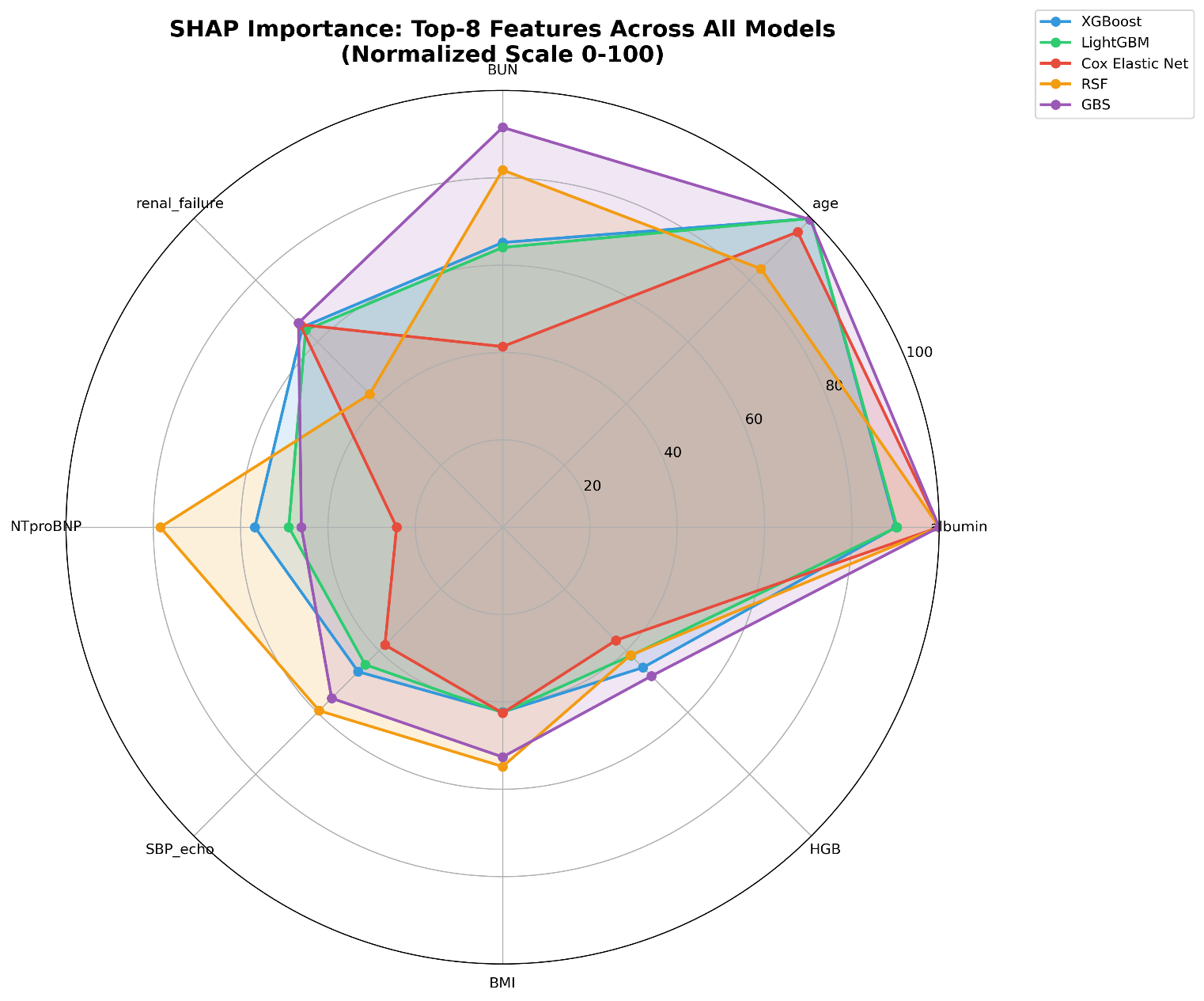


**Figure S6: Inter-model importance correlation heat-map.**


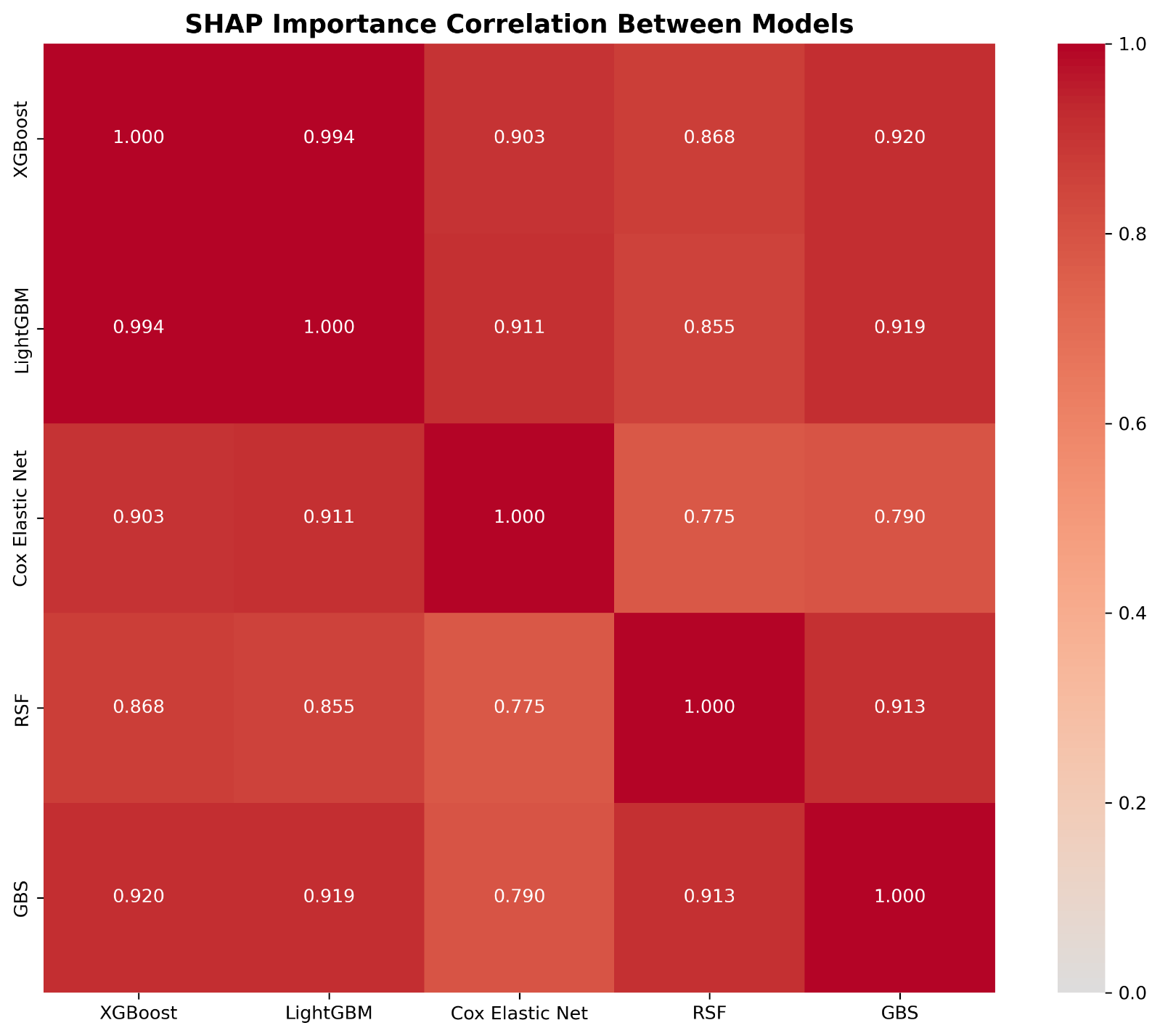


**Figure S7: Cross-model normalized comparisons.**


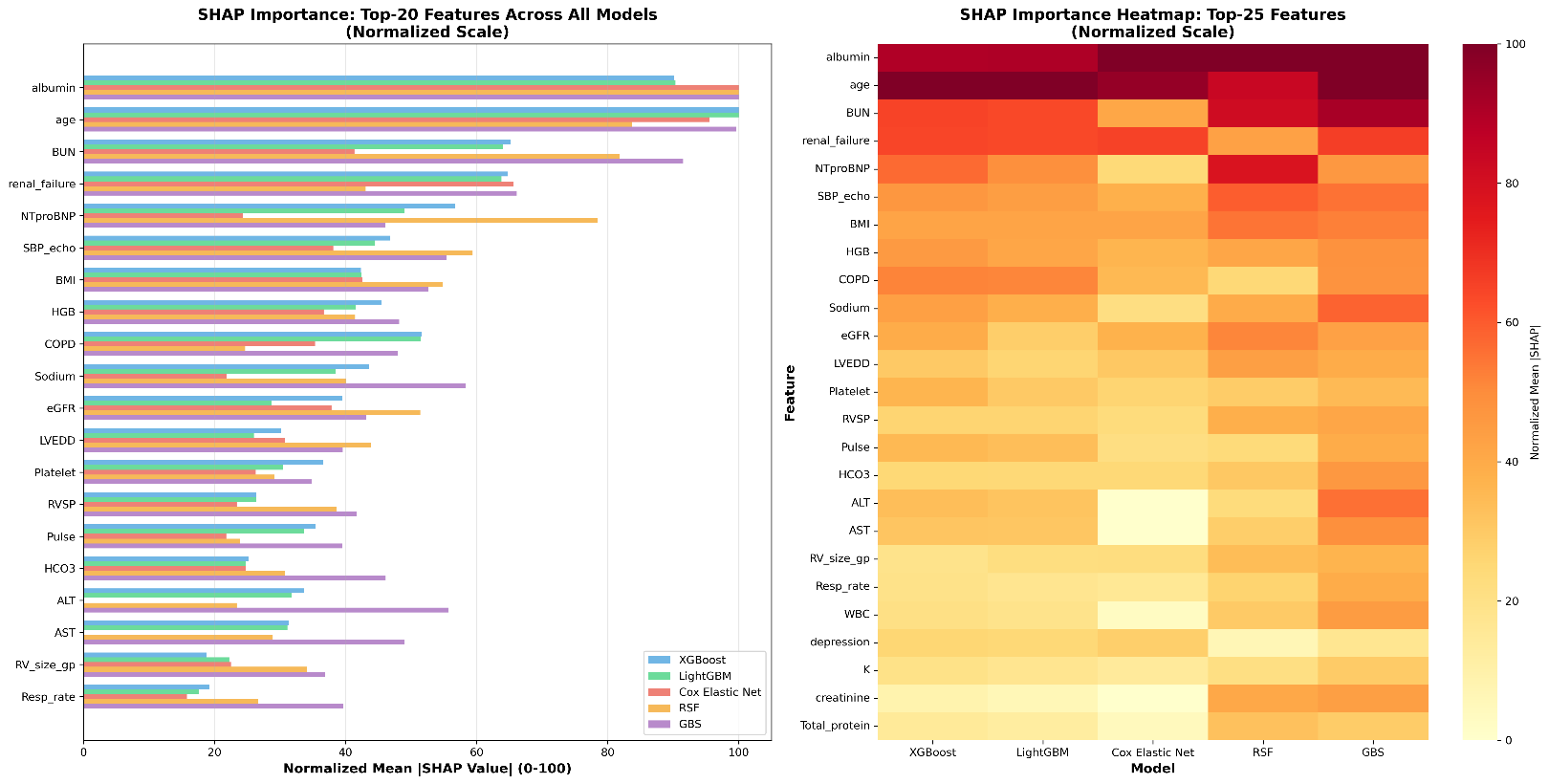


**Figure S8: XGBoost global (SHAP) explainability.**


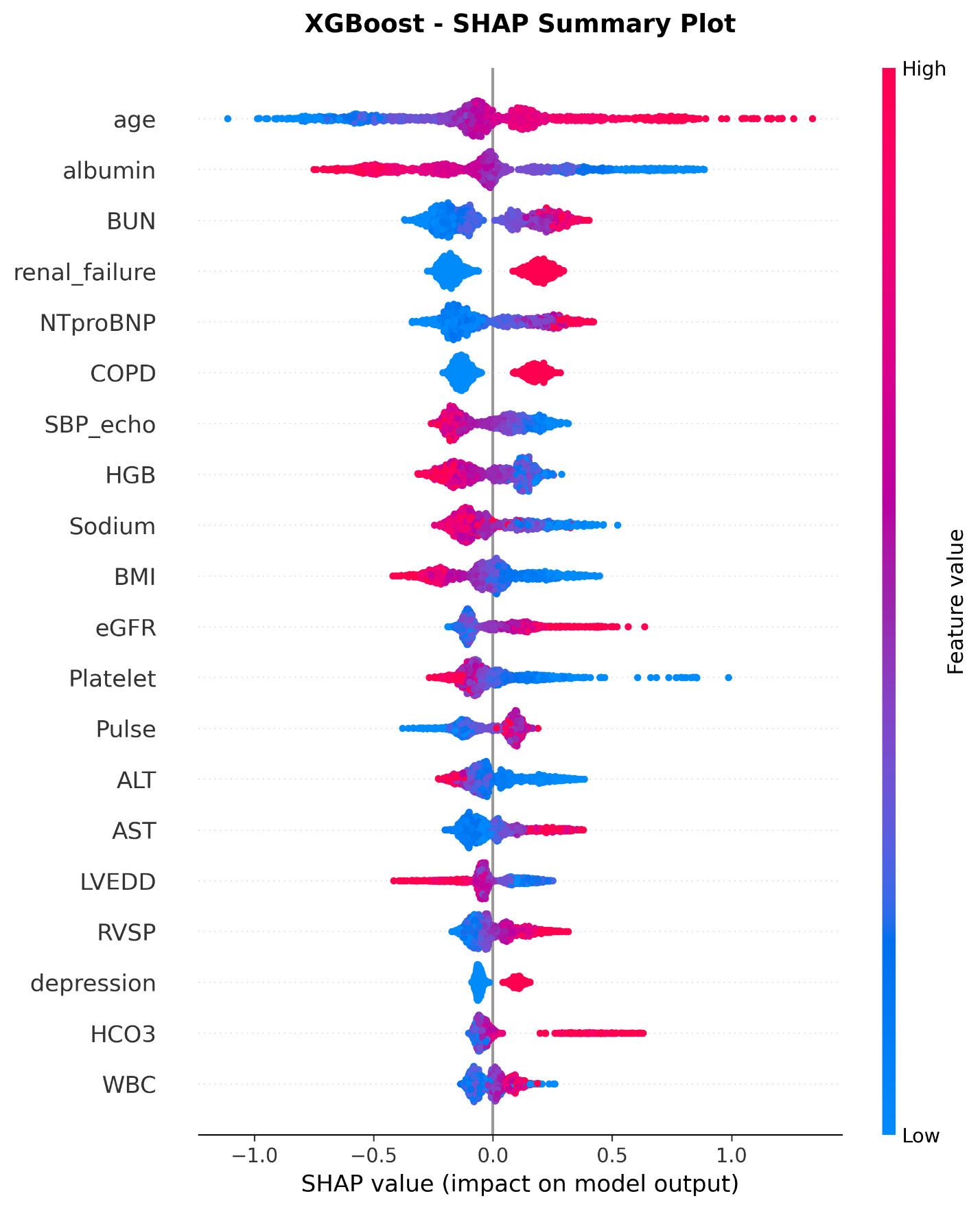


**Figure S9: LightGBM global (SHAP) explainability.**


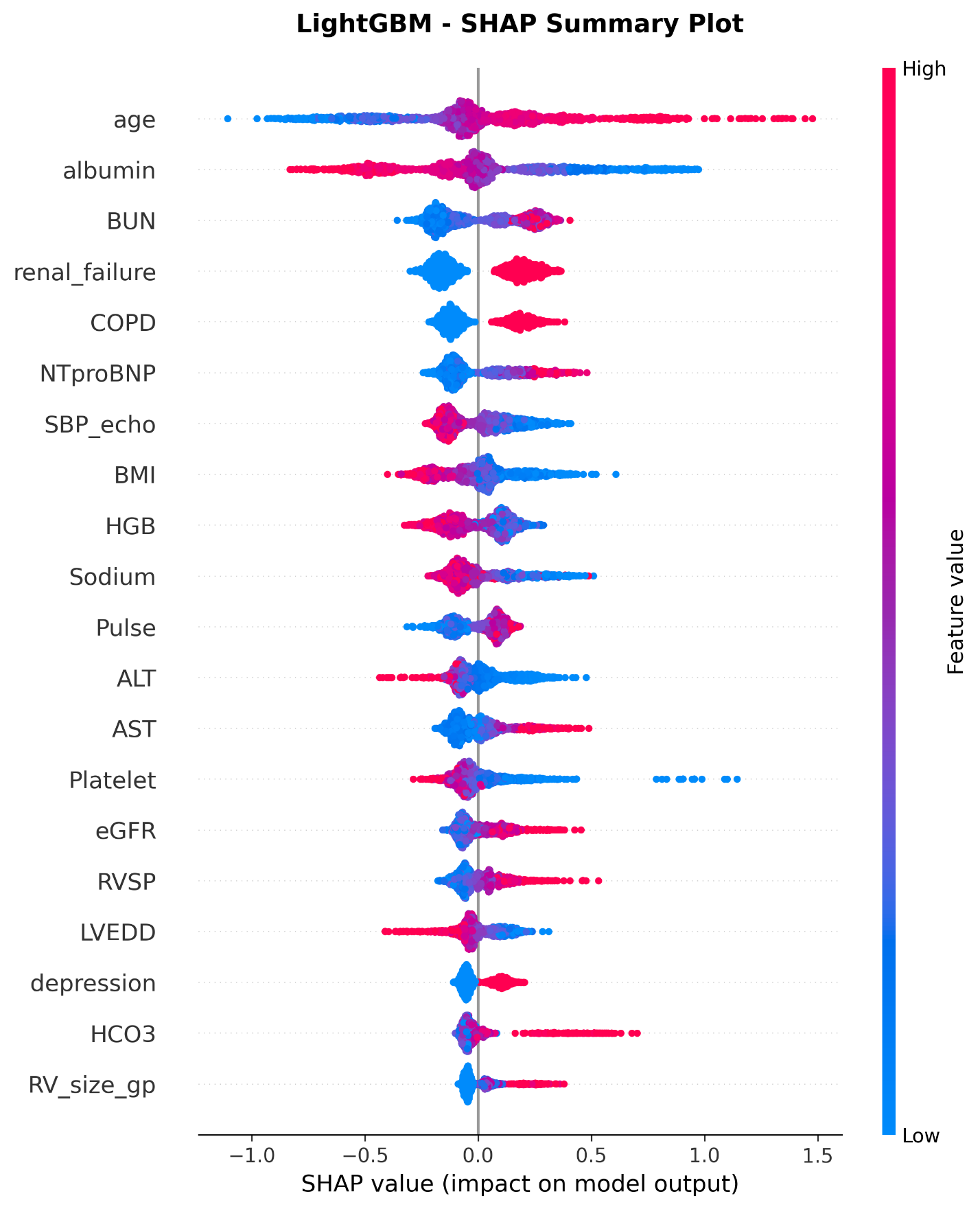


**Figure S10: Cox Elastic Net global (SHAP) explainability.**


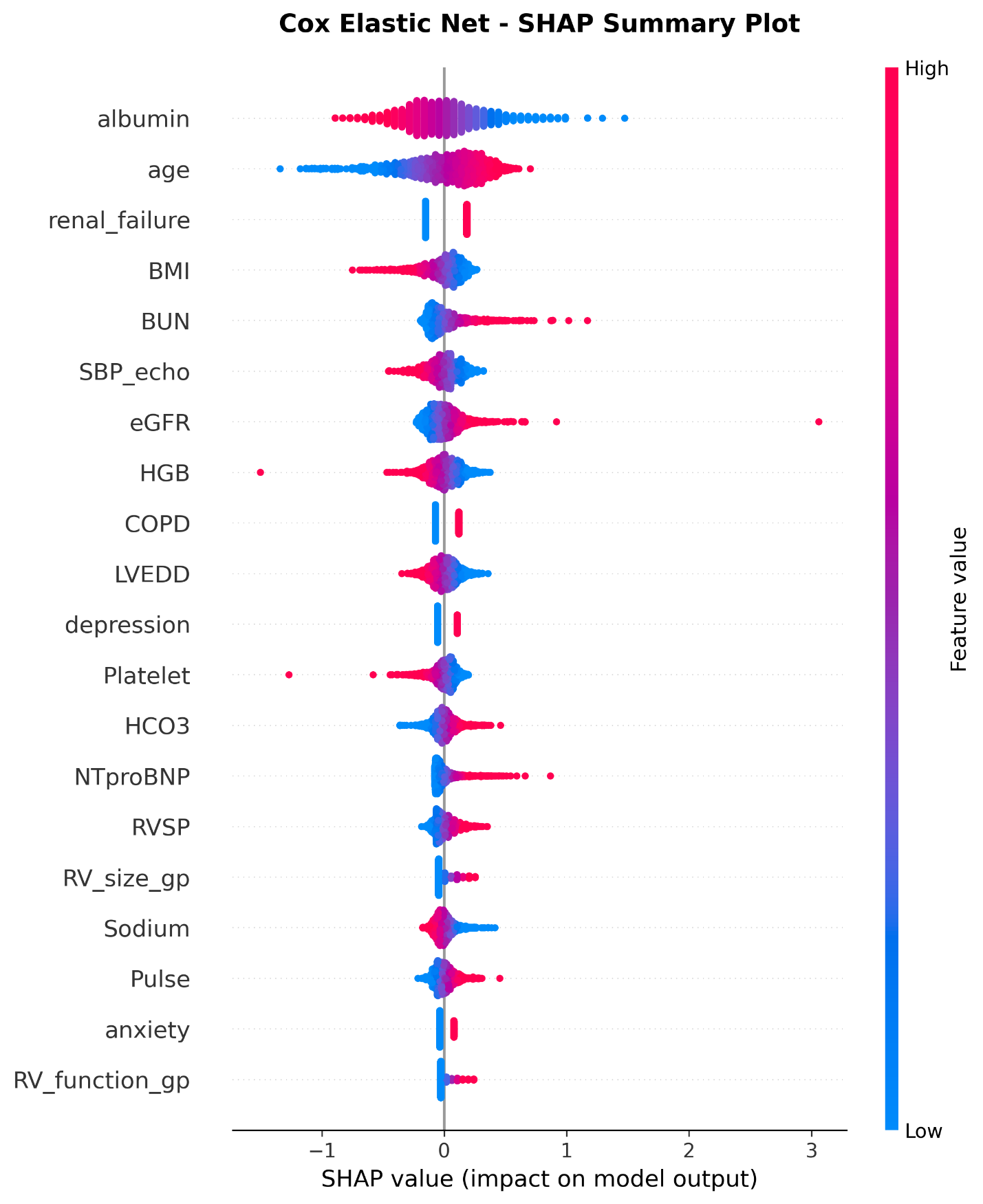


**Figure S11: RSF global (SHAP) explainability.**


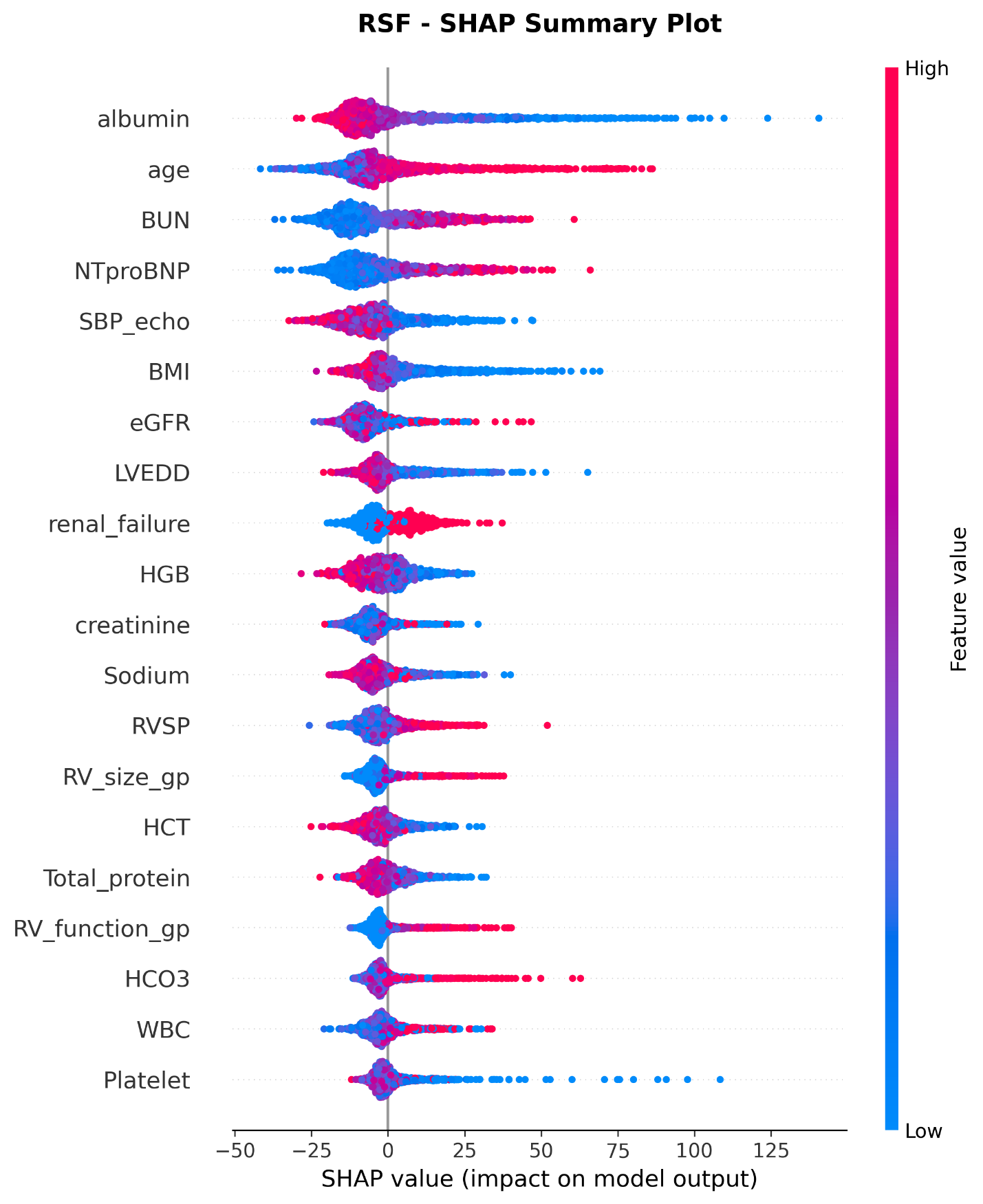


**Figure S12: GBS global (SHAP) explainability.**


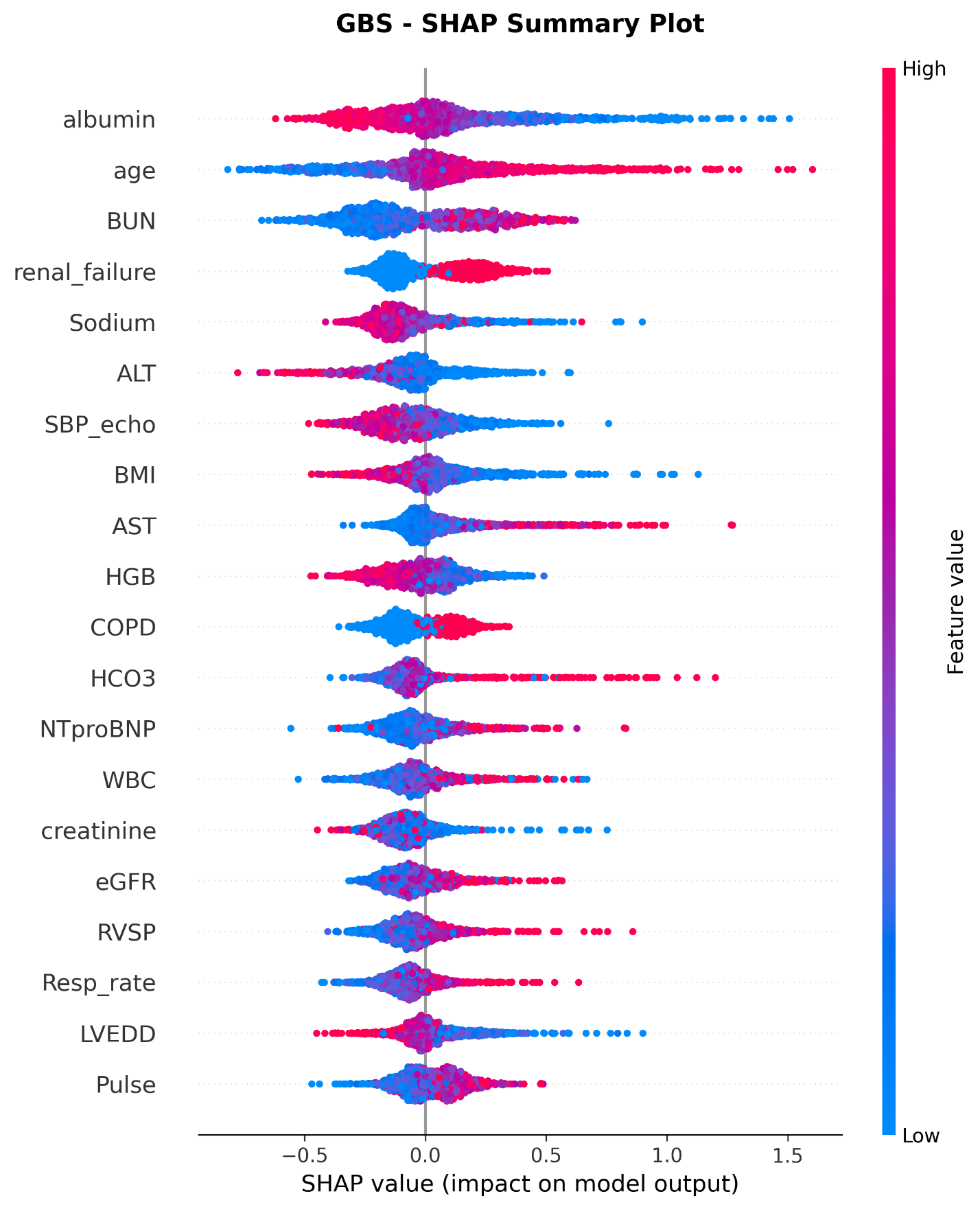


**Figure S13: XGBoost model LIME explanations (local, or patient-level).**


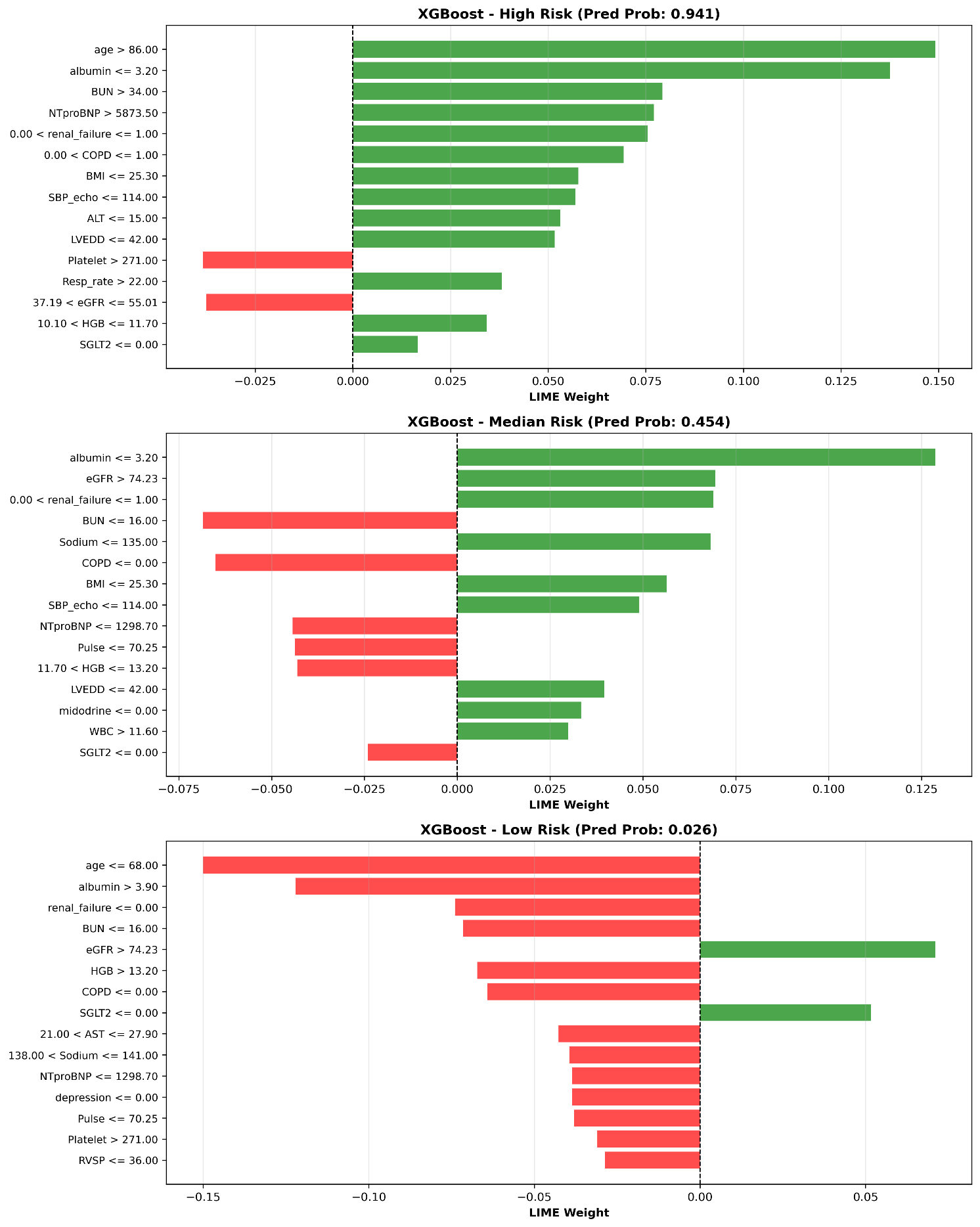


**Figure S14: LightGBM model LIME explanations (local, or patient-level).**


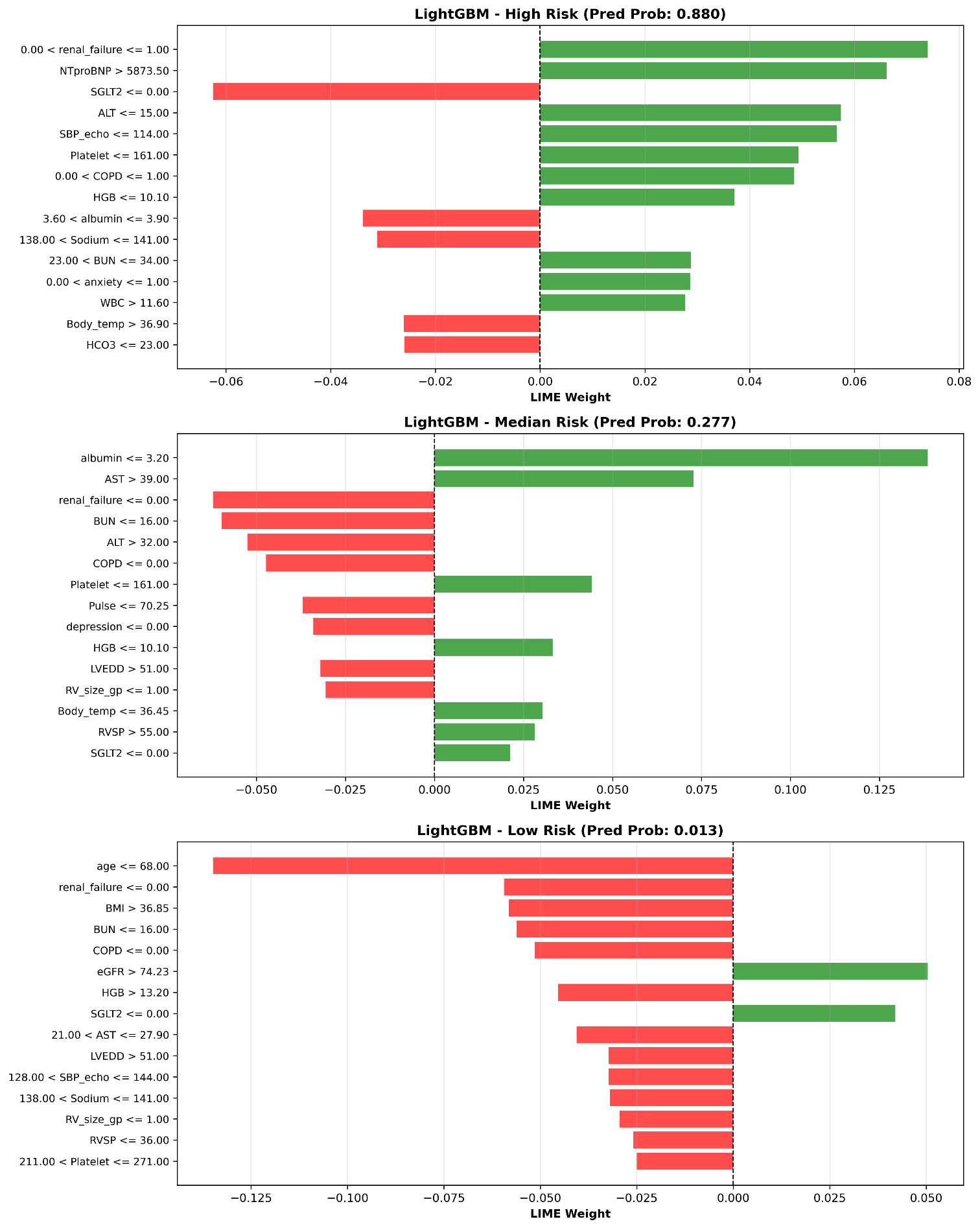


**Figure S15: Cox Elastic Net model LIME explanations (local, or patient-level).**


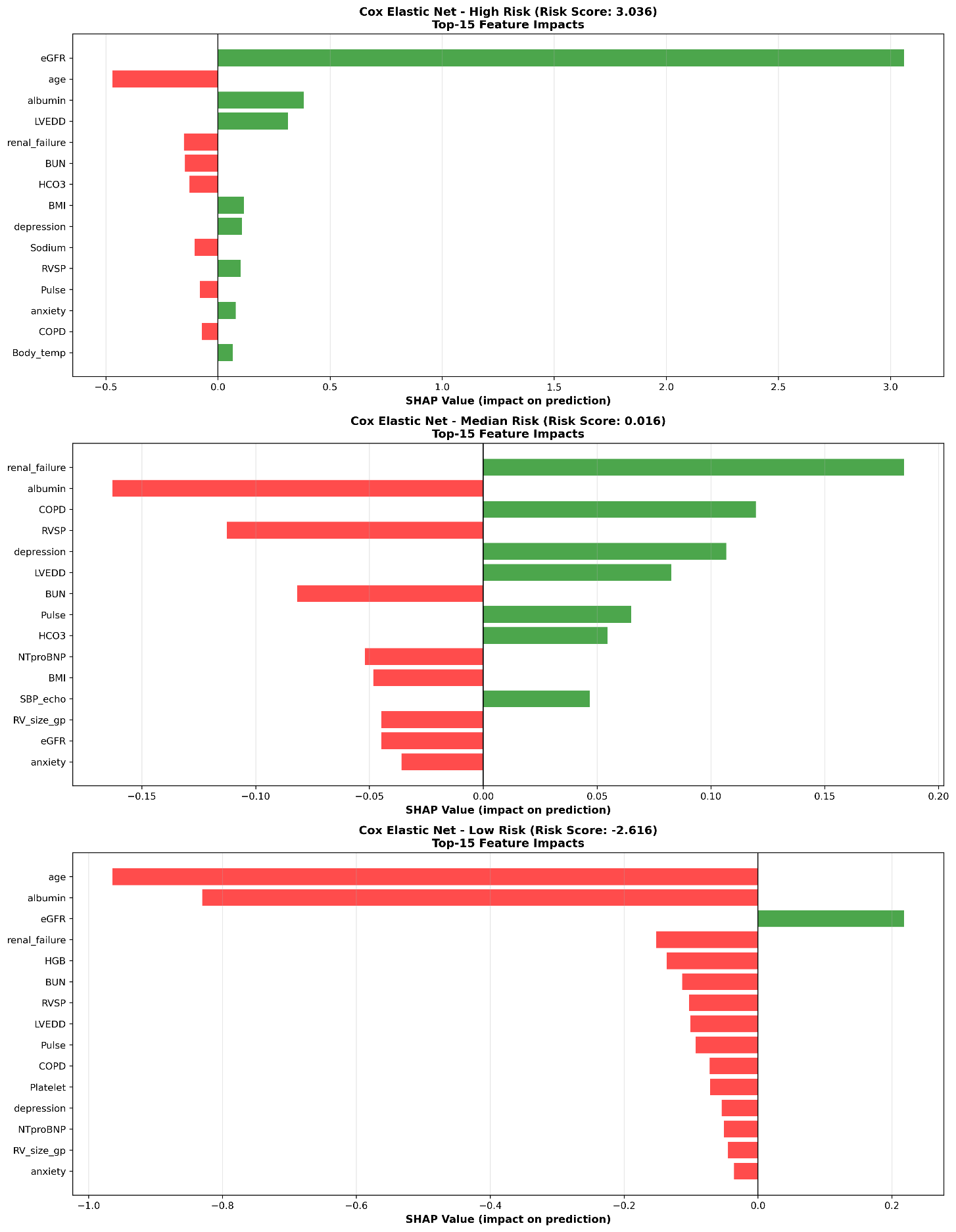


**Figure S16: RSF model LIME explanations (local, or patient-level).**


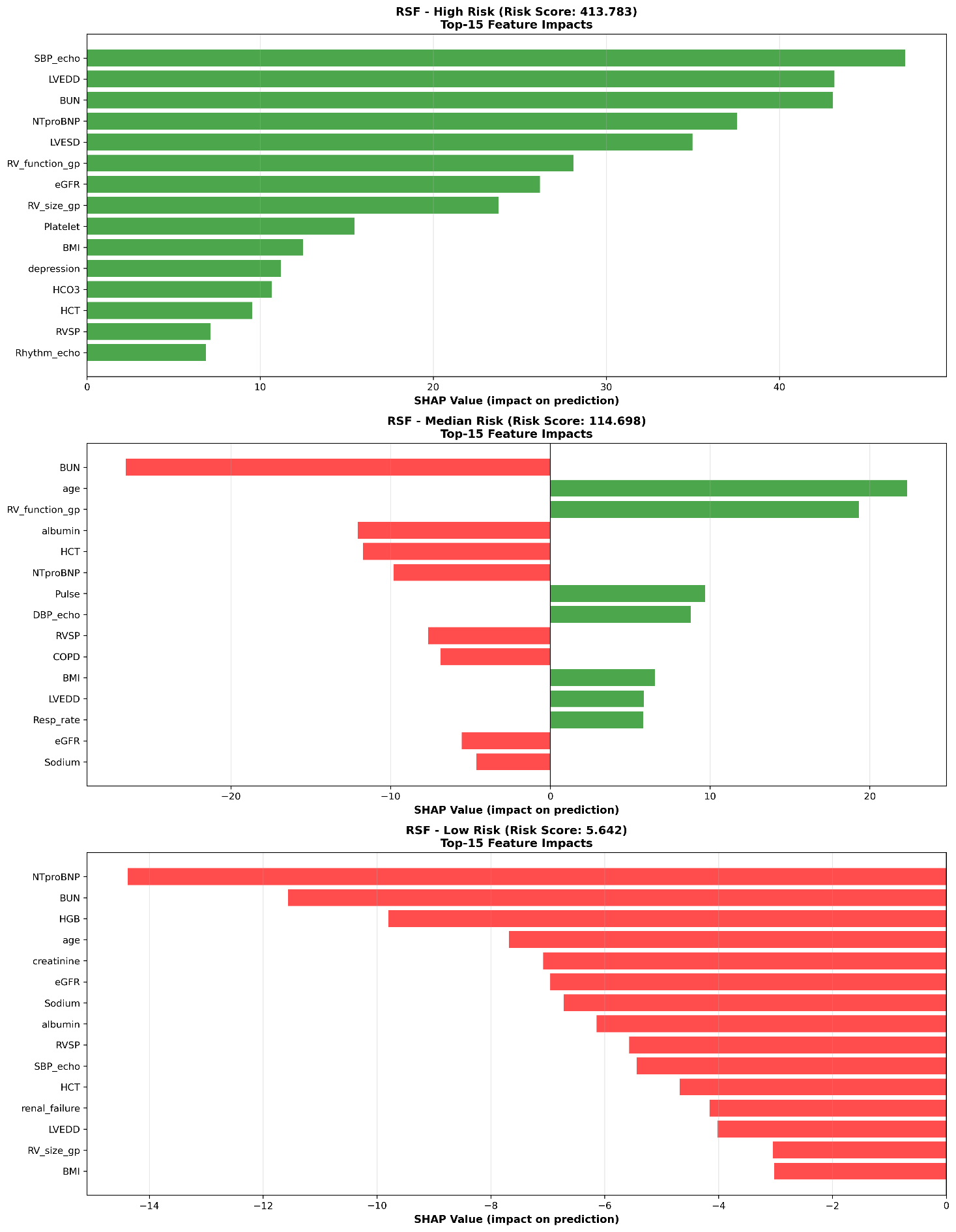


**Figure S17: GBS model LIME explanations (local, or patient-level).**


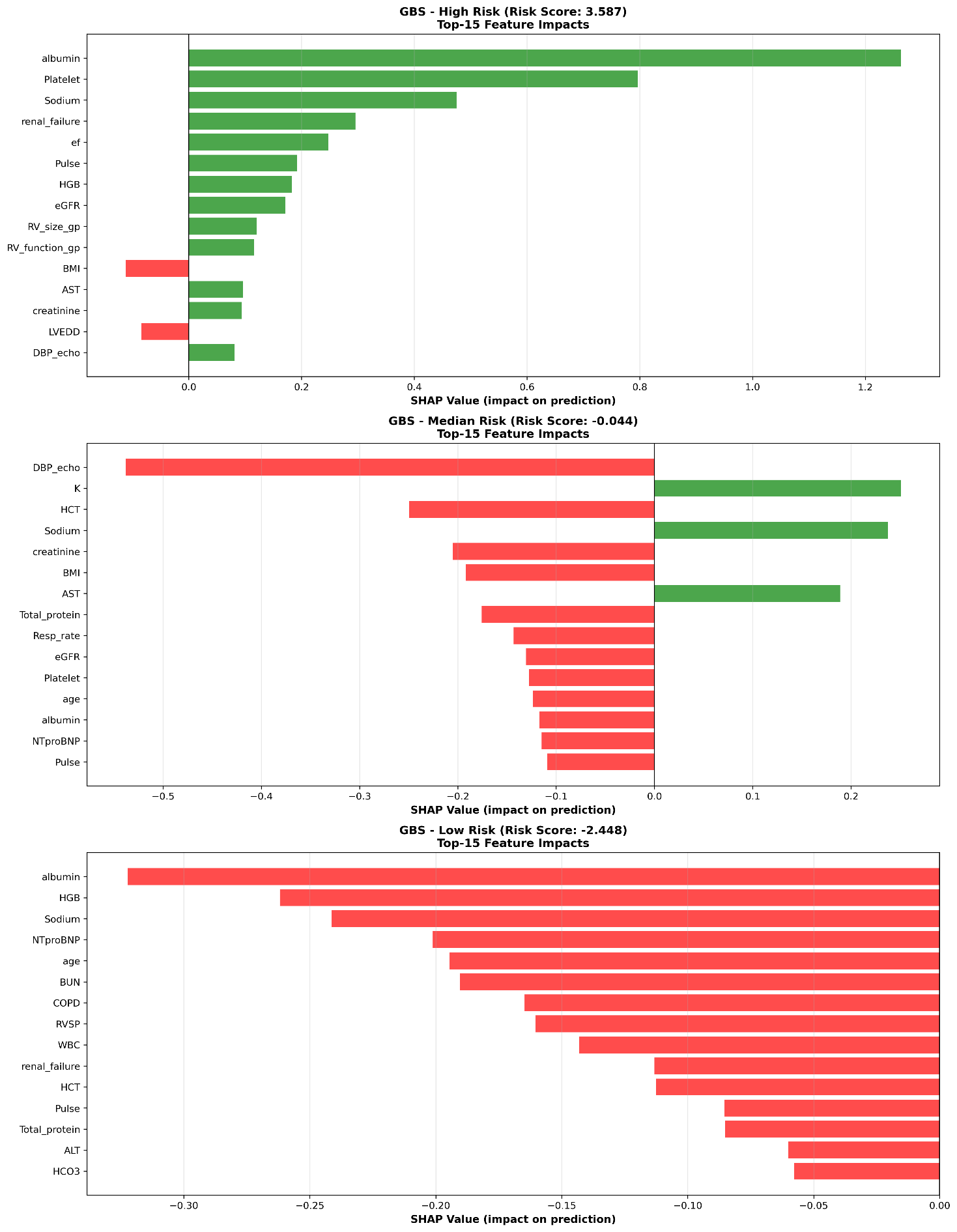

Supplement: Supplemental_Material [file mmc1.docx]
